# Mutually Orthogonal Bioorthogonal Reactions: Selective Chemistries for Labeling Multiple Biomolecules Simultaneously

**DOI:** 10.1007/s41061-024-00467-8

**Published:** 2024-07-06

**Authors:** Kevin R. Venrooij, Lucienne de Bondt, Kimberly M. Bonger

**Affiliations:** Chemical Biology Group, Department of Synthetic Organic Chemistry, Radboud University, Heyendaalseweg 135, 6525 AJ Nijmegen, The Netherlands

**Keywords:** Click chemistry, Bioconjugation, Chemical Biology, Protein Engineering, In vivo chemistry

## Abstract

Bioorthogonal click chemistry has played a transformative role in many research fields, including chemistry, biology, and medicine. Click reactions are crucial to produce increasingly complex bioconjugates, to visualize and manipulate biomolecules in living systems and for various applications in bioengineering and drug delivery. As biological (model) systems grow more complex, researchers have an increasing need for using multiple orthogonal click reactions simultaneously. In this review, we will introduce the most common bioorthogonal reactions and discuss their orthogonal use on the basis of their mechanism and electronic or steric tuning. We provide an overview of strategies to create reaction orthogonality and show recent examples of mutual orthogonal chemistry used for simultaneous biomolecule labeling. We end by discussing some considerations for the type of chemistry needed for labeling biomolecules in a system of choice.

## Introduction

There is an eminent need for new methodologies that enable precise manipulation of biomolecules to better understand the complex biological processes. An important breakthrough occurred with the development of click chemistry and bioorthogonal chemistry, which is defined as a robust set of chemical reactions that can take place within a biological system without disrupting its native biochemical processes.

The term “click chemistry” was originally introduced by Sharpless and Meldal [1, 2]. In 2001, Sharpless published a landmark article entitled: “Click chemistry: diverse chemical functionality from a few good reactions,” summarizing the essential criteria for click-type reactions [1]. Click reactions must be modular and wide in scope, while giving very high yields in a stereospecific manner and generating inoffensive byproducts [1]. “Bioorthogonal click chemistry” adds the requirement for biocompatibility, ensuring that the reactions can occur within living systems without causing harm. Bertozzi’s research group pioneered in the use of bioorthogonal chemistry in the early 2000s [3, 4], after which it quickly integrated into various research areas in chemistry, biology, engineering, and medicine and provided new opportunities to study biomolecules in complex model systems. The reactions need to be fast and robust as the physiological conditions can prove challenging owing to the presence of water and oxygen, and the click reaction often occurs under dilute conditions. Bioorthogonal click chemistry on or within cells adds further challenges, such as the presence of reactive groups in biomolecules, metals, reactive oxygen species (ROS), high concentrations of glutathione, and the low pH of endosomes and lysosomes.

The bioorthogonal toolbox is versatile, though there is a growing demand for more sophisticated bioconjugates and methods to answer increasingly complex biological questions [5–8]. Therefore, the possibility to use multiple bioorthogonal reactions simultaneously, without the occurrence of cross-reactions, is highly desired. This concept is known as “mutual orthogonal bioorthogonal chemistry”, or simply “mutual bioorthogonal chemistry.”

Applying two or more bioorthogonal reactions simultaneously in mechanistically similar pathways is possible by fine-tuning the steric and electronic properties of the reactants. Introducing substituents that alter the steric or electronic properties on the reactive groups can increase or decrease the reaction kinetics by several orders of magnitude [9]. An additional layer of orthogonality can be installed by inducing proximity of slow-reacting reaction partners or by using an external trigger to initiate the reaction, such as light.

In this review, we will provide an overview of mutual orthogonal chemistry, focusing on its principles, applications, and recent advancements. We will first cover the most prevalent click reactions, examine the opportunities for mutual orthogonal chemistry and provide guidelines on how to apply these reactions in complex chemical systems as well as living systems. In this context, we discuss the tuning of the electronic properties by introducing electron-withdrawing (EWG) or electron-donating groups (EDG) and discuss the influence of steric substituents. We will then highlight the potential for activation using external light triggers and proximity enhanced click chemistry to capitalize on slower kinetic reactions under dilute conditions. Finally, we provide some examples of using multiple reactions in bioconjugation reactions and discuss some challenges in the field.

## Types of Click Chemistry

**Fig. 1:**
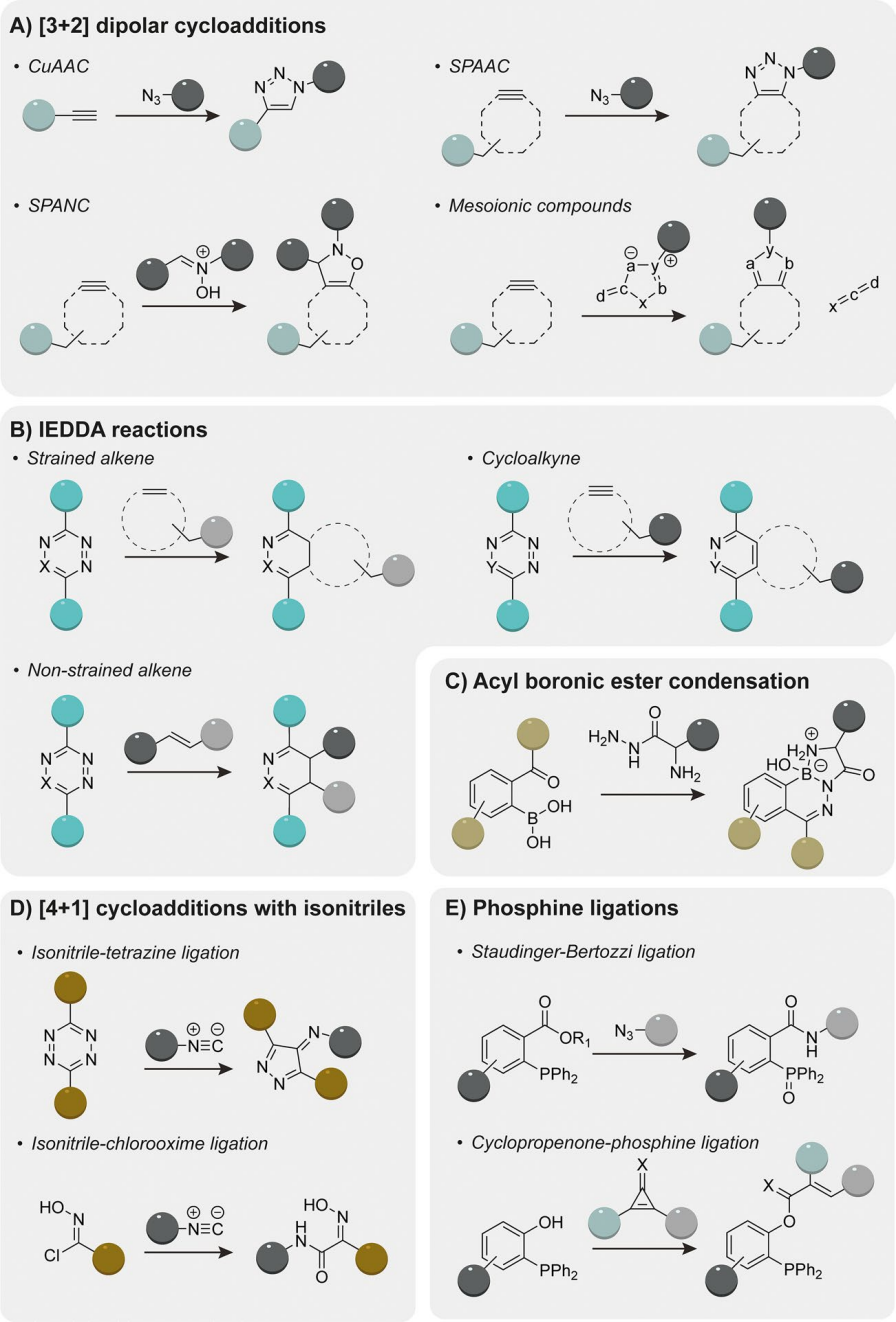
Schematic representation of bioorthogonal reactions often used in bioconjugation. **A** [3 + 2] dipolar cycloadditions. (i) copper-catalyzed azide-alkyne cycloaddition (CuAAC), (ii) strain-promoted azide-alkyne cycloaddition (SPAAC), (iii) strain-promoted alkyne-nitrone cycloaddition (SPANC), and (iv) mesoionic compounds. **B** Inverse electron demand Diels–Alder reaction. (i) IEDDA with strained alkene, (ii) IEDDA with cycloalkyne, and (iii) IEDDA with non-strained alkene. **C** Boronic ester condensation. **D** [4 + 1] cycloaddition with isonitrile. (i) Isonitrile-tetrazine ligation and (ii) isonitrile-chlorooxime ligation. **E** Phosphine ligations. (i) Staudinger-Bertozzi ligation and (ii) cyclopropenone-phosphine ligation

Over the last two decades, many bioorthogonal reactions have been reported and tailored to the application and biological system used. In here, we will focus on five types of click reactions often used in bioconjugations as shown in Fig. 1 namely, (A) [3 + 2]-dipolar cycloadditions, (B) inverse electron-demand Diels–Alder reactions, (C) phosphine ligations, (D) [4 + 1] isonitrile cycloadditions, and (E) boronic ester condensations. We also discuss their potential for (sequential) orthogonal use in bioconjugations in vitro or within living cells.

### [3 + 2] Dipolar Cycloadditions

#### Copper-Catalyzed Azide-Alkyne Cycloadditions (CuAAC)

The [3 + 2] dipolar cycloaddition involves the interaction between a 1,3-dipole and an alkyne or alkene dipolarophile, resulting in the formation of a five-membered heterocycle (Fig. 1A). While uncatalyzed reactions with non-strained alkynes proceed only at elevated temperatures, the regioselective copper (I)-catalyzed azide-alkyne cycloaddition (CuAAC) occurs at ambient temperatures. Sharpless and Meldal were the first to independently report the use of this reaction in a biocompatible setting more than two decades ago [2, 10]. Alternative catalysts for the azide alkyne cycloadditions have also been reported. Ruthenium (II) complexes catalyze the cycloaddition, but compared with the CuAAC reaction that yields the 1,4-triazole isomer, the ruthenium-catalyzed AAC (RuAAC) is slower and yields the 1,5-isomer [11–13] Despite efforts to reduce the cellular toxicity of the metal species using stabilizing ligands [14], the use of the metal-catalyzed azide-alkyne cycloadditions in living systems is not recommended.

#### Strain-Promoted Azide-Alkyne Cycloadditions (SPAAC)

In 2004, Bertozzi introduced the bioorthogonal strain-promoted azide-alkyne cycloaddition (SPAAC) as an alternative to CuAAC (Fig. 1A) [15]. The sp-hybridized alkynyl carbons cause high ring strain on the cyclic alkynes, bending the alkyne from its normal linear geometry. Since the release of ring strain drives the cycloaddition reaction, there is no copper catalyst required, making the SPAAC more suitable for use in living systems. Yet, compared to the CuAAC reaction, the SPAAC reaction holds a significant kinetic disadvantage in [3 + 2]-cycloadditions.

**Fig. 2:**
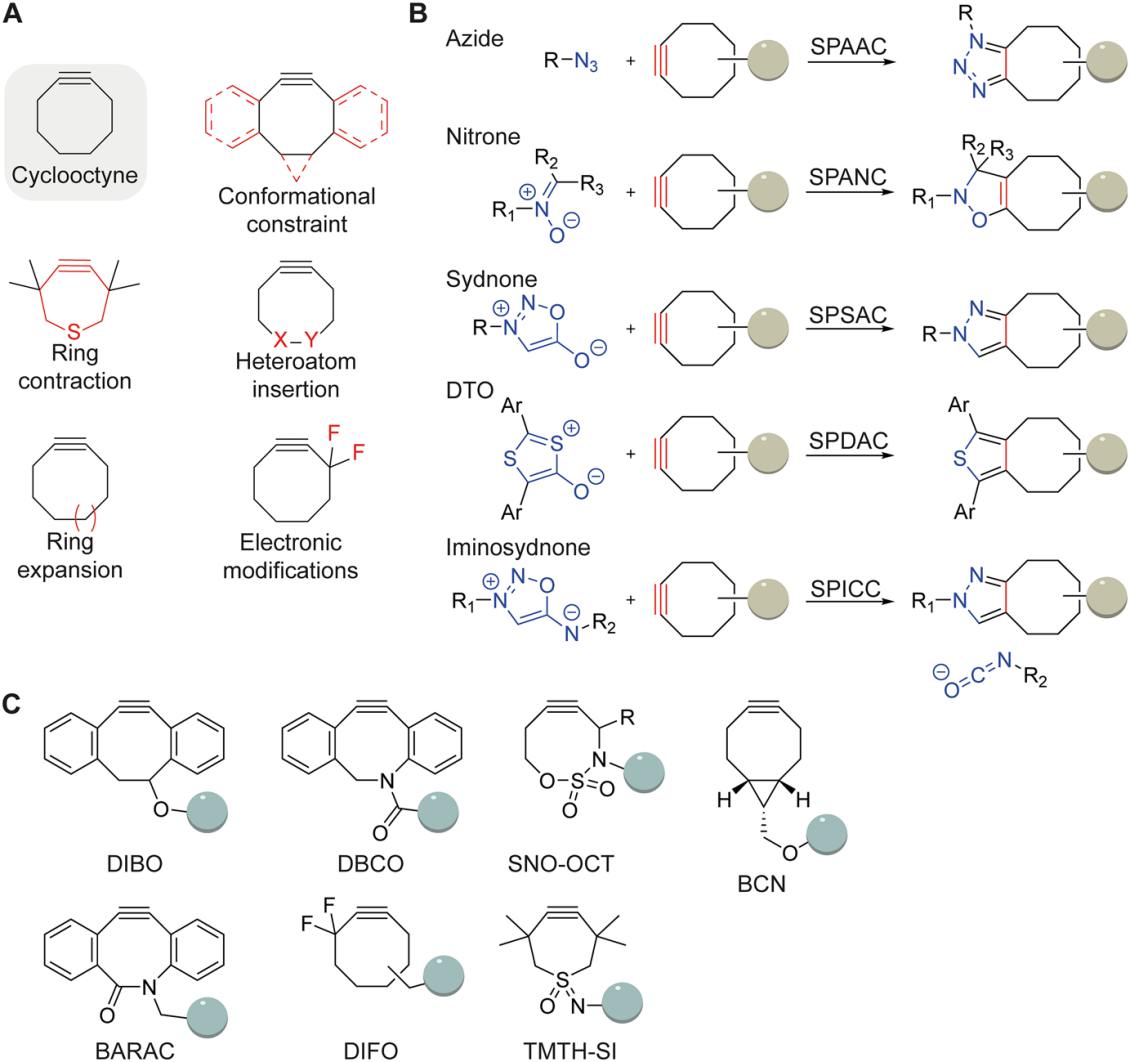
**A** Strategies to increase reaction rates of strained alkynes. Conformational constraint, ring contraction, heteroatom insertion, ring expansion and electronic modifications [28]. **B** Reactions of azide, nitrones, and mesoionic heterocycles with cyclooctynes. **C** Overview of the structures of common strained alkynes

To increase the rates of the SPAAC reaction, researchers investigated modifications of the ring structure including size, conformation, electronic modifications, and inclusion of heteroatoms (Fig. 2A and C). For example, Bertozzi and colleagues showed that a *gem*-difluoro group on the propargylic position electronically activates the alkyne in their difluorinated cyclooctyne (DIFO) reagents [16]. Alternatively, increased ring strain is possible with fused rings, such as bicyclo[6.1.0]non-4-yne (BCN) or 4-dibenzocyclooctynol (DIBO) [17]. Inclusion of a sp^2^-like nitrogen center increases further enhances the reaction rate, such as biarylazacyclooctynones (BARAC) [18] and aza-dibenzocyclooctynes (DIBAC/DBCO) [19]. The additional aromatic rings present in diarylcyclooctynes, however, make the molecule bulky and less water soluble, which may be unfavorable for some applications.

The less bulky tetramethylthiacycloheptyne (TMTH), a seven-membered ring with an endocyclic sulfur atom, has faster kinetics than DIFO owing to increasing the strain energy [20]. The more reactive reactants, including BARAC and TMTH, are less stable and more likely to undergo side reactions [21]. For example, BARAC undergoes hydrolysis in phosphate buffered saline (*t*_1/2_ 24h) and an intramolecular rearrangement may occur under acidic conditions [22]. In general, cyclooctynes are susceptible to nucleophilic addition with reactive cysteines or glutathione present in the intracellular environment [23]. Other side reactions, such as homotrimerization [24] and reactions with cysteine sulfenic acid, may also occur [25]. As TMTH lacks a convenient point for ligand attachment [26], a more stable TMTH-sulfoximine (TMTH-SI) variant was recently reported allowing for ligand conjugation. TMTH-SI additionally reacts somewhat faster with 1,3-dipoles compared with DBCO [27].

#### Alternative Cycloadditions Beyond Azides

Besides azides, other 1,3 dipoles have been extensively explored in reactions with strained alkynes. Most notably are the reactions with nitrones and mesoionic heterocycles.

The allyl-type nitrone dipole forms the stable *N*-alkylated isoxazoline product when reacted with a cytooctyne in the strain-promoted alkyne-nitrone cycloaddition (SPANC; Fig. 2B) [29–31]. Nitrones are biologically stable and show rapid cycloaddition kinetics. Contrary to azides, nitrones contain three sites for modification that allow for manipulation, making them highly tunable dipoles for bioorthogonal cycloadditions.

Diazo compounds can undergo [1 + 3] cycloaddition with strained alkynes [32]. Their reactivity depends on the conjugation of flanking groups, which can result in improved kinetics over azides [33]. Moreover, [1 + 3] cycloadditions between diazo and alkenyl compounds can occur selectively in the presence of an azide [34]. However, diazo compounds readily react with carboxylic acids, which is a major downside for applications in the bioorthogonal context [35].

Mesoionic heterocycles, such as sydnones act as 1,3 dipoles owing to their delocalized charge distribution. Identified in a high throughput screening campaign, Taran et al. reported on the biocompatible copper-catalyzed cycloaddition between sydnone and nonstrained alkynes [36]. The strain-promoted sydnone-alkyne cycloaddition (SPSAC) was reported soon after by Chin’s group and occurs via the [3 + 2]-cycloaddition, followed by a retro-Diels–Alder, forming a pyrazole and CO_2_ (Fig. 2B) [37, 38]. The SPSAC reaction rate is comparable to the SPAAC, but slower than the SPANC and CuAAC. Taran et al. introduced the strained-promoted iminosydnones cyclooctyne cycloaddition (SPICC), which involves iminosydnones and cycloalkynes and generates a pyrazole click product, releasing an isocyanate (Fig. 2B) [39, 40]. Although reaction rates are rather slow, this class of compounds allows the release of cargo upon cycloaddition reaction [39, 40]. The strain-promoted 1,3-dithiolium-4-olate/alkyne cycloaddition (SPDAC) requires the sulfur-based family of 1,3-dithiolium-4-olates (DTOs) mesoionics, and reacts in the same order of magnitude as SPSAC (Fig. 2B) [41]. Interestingly, owing to the rather bulky structure, DTOs react with BCN but do not react with the more bulky DBCO cyclooctyne.

#### Tuning Reactivity within [3 + 2] Dipolar Cycloadditions

**Fig. 3:**
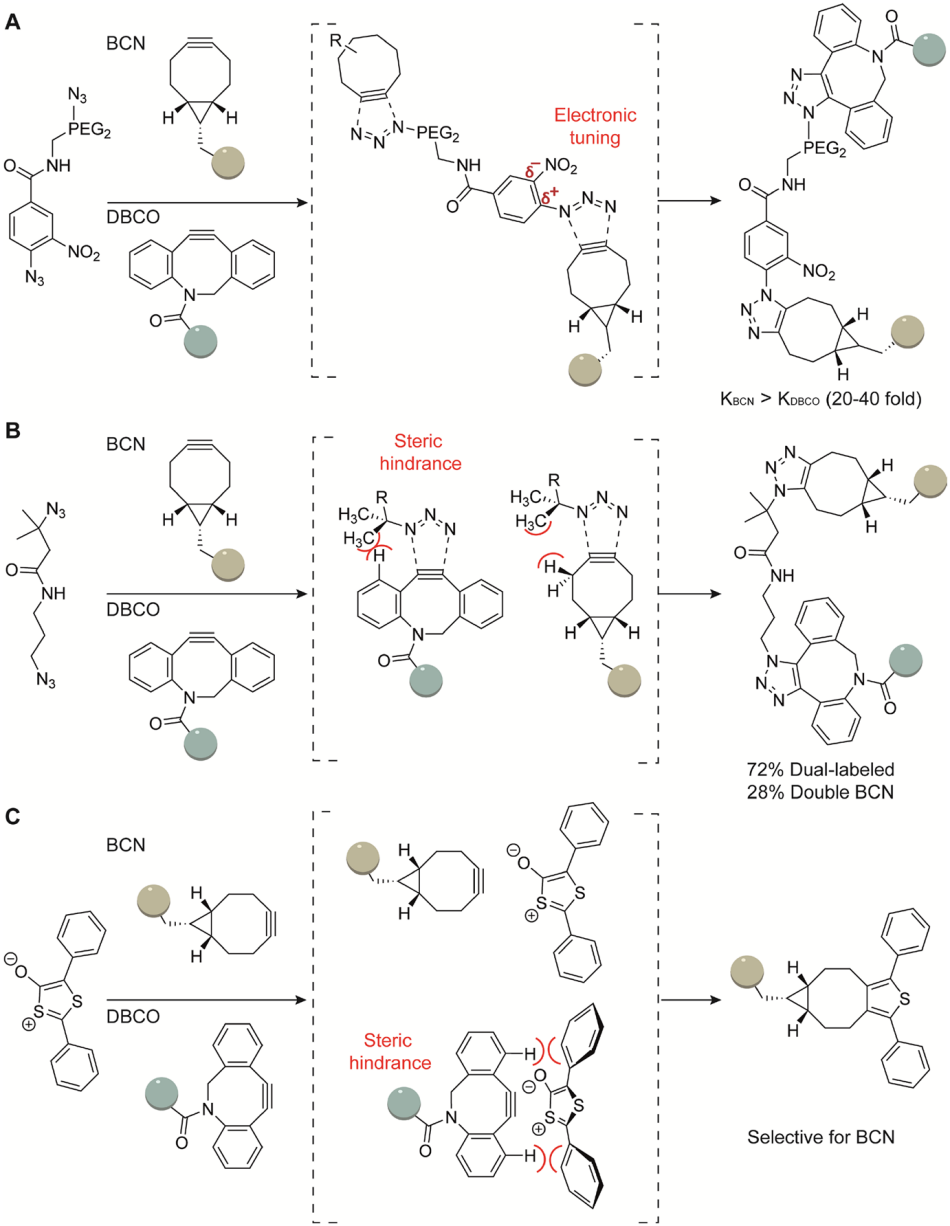
Mutual orthogonal labeling through steric tuning. **A** Inverse-electron demand (IED) mechanism occurs with BCN but not DBCO. **B** A duel-labeling experiment with two SPAAC reaction. DBCO-SiR preferentially reacts with the primary azide while BCN-BODIPY can react with the remaining tertiary azide [43]. **C** DTO reacts with BCN but not DBCO owing to steric hindrance

The type of mechanism that drives the cycloaddition is dictated by the electronic properties of the reactants. Bicycle[6.1.0]nonyne (BCN), a cyclooctyne with a fused cyclopropane ring, is less bulky compared with DBCO [42]. Van Delft demonstrated that benzyl azides react faster with DBCO compared with BCN. The opposite is true for phenyl azides, that react faster with BCN [21]. DFT calculations revealed that BCN reacts with phenyl azides via an inverse-electron demand (IED) mechanism [21]. In here, the reaction is driven by the difference between HOMO_BCN_ and LUMO_azide_, compared with most SPAAC reactions that follow interaction of HOMO_azide_ and LUMO_cyclooctyne_. This observation found that the use of electron poor azides in reactions with BCN boost the reaction rate by at least one order of magnitude [21]. Van Delft further exploited this unique reactivity difference in a single pot orthogonal labeling of a BCN-labeled protein and a DBCO-modified fluorescent dye to a linker equipped with both an electron poor azide and an aliphatic azide (Fig. 3A).

Besides altering electronic properties, bulky reactants can alter the reactivity within the [3 + 2] cycloaddition. Mikula reported the chemoselective SPAAC reaction of BCN with tertiary azide in the presence of the sterically demanding DBCO with either a primary or secondary azide [43]. They applied the distortion/interaction model developed by Bickelhaupt and Houk, describing that steric hindrance increases distortion energy, thereby decreasing reactivity [44]. For example, DBCO reacts ~ 19-fold slower with tertiary azides compared with primary azides. The less bulky BCN reacts twofold slower with tertiary azides. Therefore, sequential orthogonal labeling is possible with DBCO reacting with the primary azide in the first step, and subsequently adding BCN that reacts with the tertiary azide (Fig. 3B). When the reagents are added simultaneously, the yield of the desired bifunctional labeled product is reduced to 72%, with the remaining 28% being double BCN labeled [43].

Similarly, owing to the bulky phenyl substituents, DTO preferentially reacts with less sterically demanding BCN compared with DBCO. In a double-click experiment, Taran and coworkers showed that SPDAC with DTO and BCN followed by SPAAC with an azide and DBCO could be performed orthogonally by preventing cross-reactivity owing to steric interference. (Fig. 3C) [41].

### Inverse Electron Demand Diels–Alder Cycloaddition (IEDDA) Reactions

#### Dienes

The inverse electron-demand Diels–Alder (IEDDA) reaction between an electron-poor diene and electron-rich dienophile has grown to one of the most commonly used reactions for bioconjugations (Fig. 1B). This reaction forms a six membered ring in a π4s + π2s mechanism, where the 4π-electrons of the diene interact with the 2π-electrons of the dienophile [45]. Tetrazines are often used as diene, while the dienophiles are rather diverse and include cis or trans (strained) alkenes or alkynes. The reaction kinetics are commonly dictated by the energy gap between the LUMO_diene_ and HOMO_dienophile_. Consequently, substituting the tetrazine diene with electron deficient groups lowers the LUMO energy and thereby increases its reactivity [45].

**Fig. 4:**
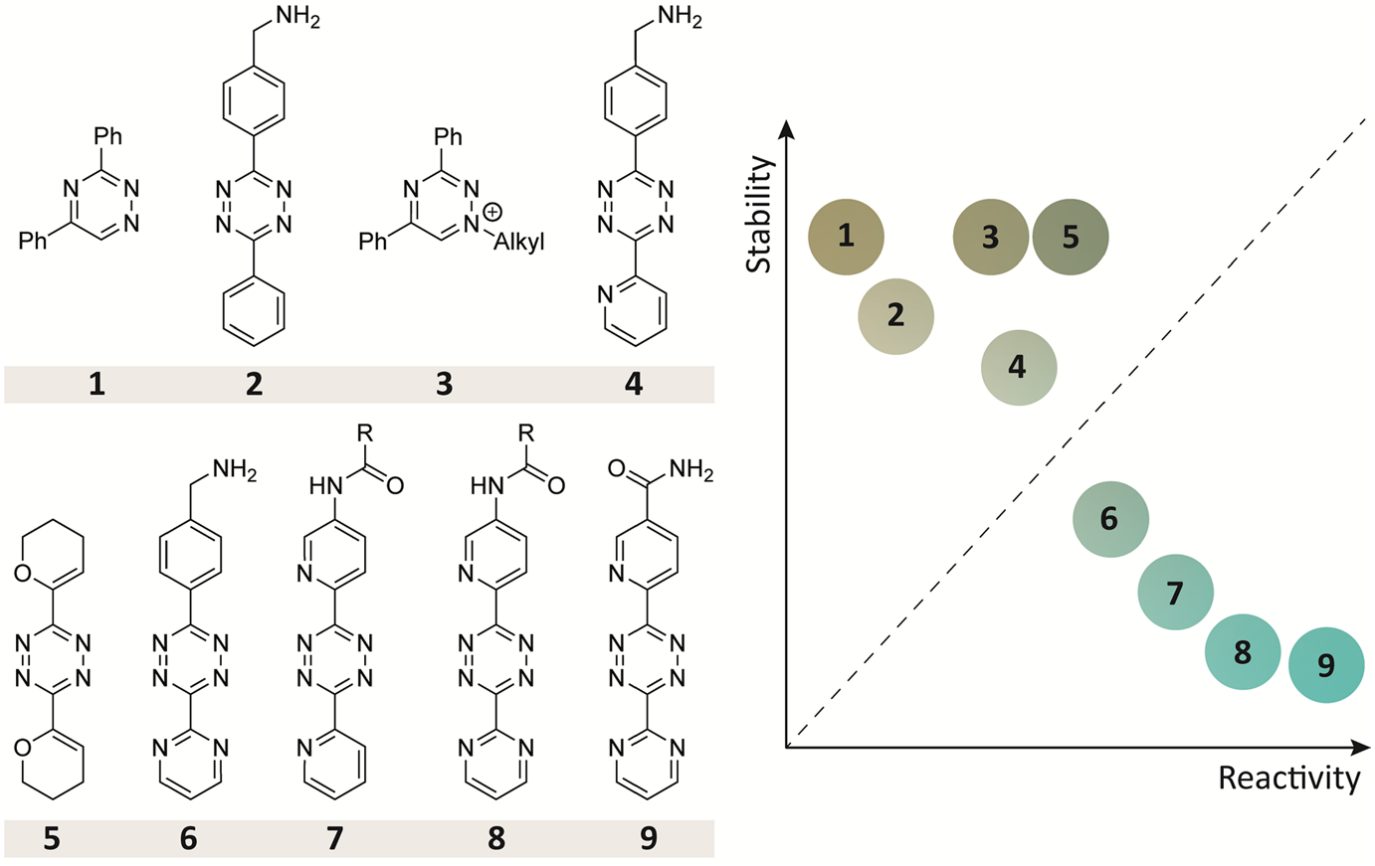
The reactivity of tetrazine scaffolds based on the electronic effect of the substitution pattern. Electron-withdrawing groups increase the reactivity and kinetics. Tetrazines with increased reactivity show decreased stability under physiological conditions [46–50]

Since its introduction by the Fox’s group in 2008, the IEDDA reaction has gained a lot of attention owing to its superior reaction kinetics, rendering it exceptionally suitable for in vivo applications [51]. Altering the electron density of the tetrazines by varying the substitutions at the C3 and C6 positions has led to a wide range of 1,2,4,5-tetrazine derivatives with diverse reactivities (Fig. 4). Electron-withdrawing substituents increases tetrazine reactivity, yet, they are also more susceptible to nucleophilic attack and, consequently, degradation in cell culture media [45, 48, 52, 53]. For example, the dipyridyl tetrazine reacts extremely fast with TCOs, but less than 1% is still intact after 24 h in cell growth medium [53]. Mikula et al. demonstrated that the high reactivity of 2-pyridyl substituted tetrazines was additionally attributed to the intramolecular repulsive N–N interaction rather than sole electron withdrawing properties of the tetrazine substituents. Conversely, more stable tetrazines were developed containing electron donating dihydro-2H-pyran (DHP) substituent that shows fast reaction rates owing to the distortion between the oxygen and nitrogen of the tetrazine [53].

1,2,4-Triazines are more stable in biological conditions compared with tetrazines. However, these reagents demonstrate several orders of magnitude slower reaction kinetics than tetrazines [54]. Other azabenzenes, including 1,3,5-triazines, 1,2-diazines, and 1,2,3-triazines, have been studied in IEDDA reactions but improved stability resulted in reduced reaction rates [55].

An interesting new class of compounds reported by the group of Vrabel are the N1-alkyl-1,2,4-triazinium salts that display orders of magnitude faster reaction rates with BCN compared with unsubstituted triazines [56]. The authors demonstrate good stability, solubility, and could be used for intracellular protein labeling. In addition, the triazonium salts do not react with DBCO making them orthogonal to the SPAAC reaction [56].

#### Dienophiles

In IEDDA reactions, the reactivity of the dienophiles depends on the strain as well as the electronic properties where: (1) strained dienophiles are more reactive than nonstrained counterparts, (2) electron-rich substituents improve reaction kinetics, and (3) an increase of steric hindrance lowers the reaction kinetics. Strained alkenes, especially trans-cyclooctenes (TCO), are the most commonly used dienophiles. The strain-promoted inverse electron demand Diels–Alder (SPIEDAC) between TCO and tetrazine is the fastest IEDDA reaction owing to the high ring strain introduced by the trans double bond [45].

**Fig. 5:**
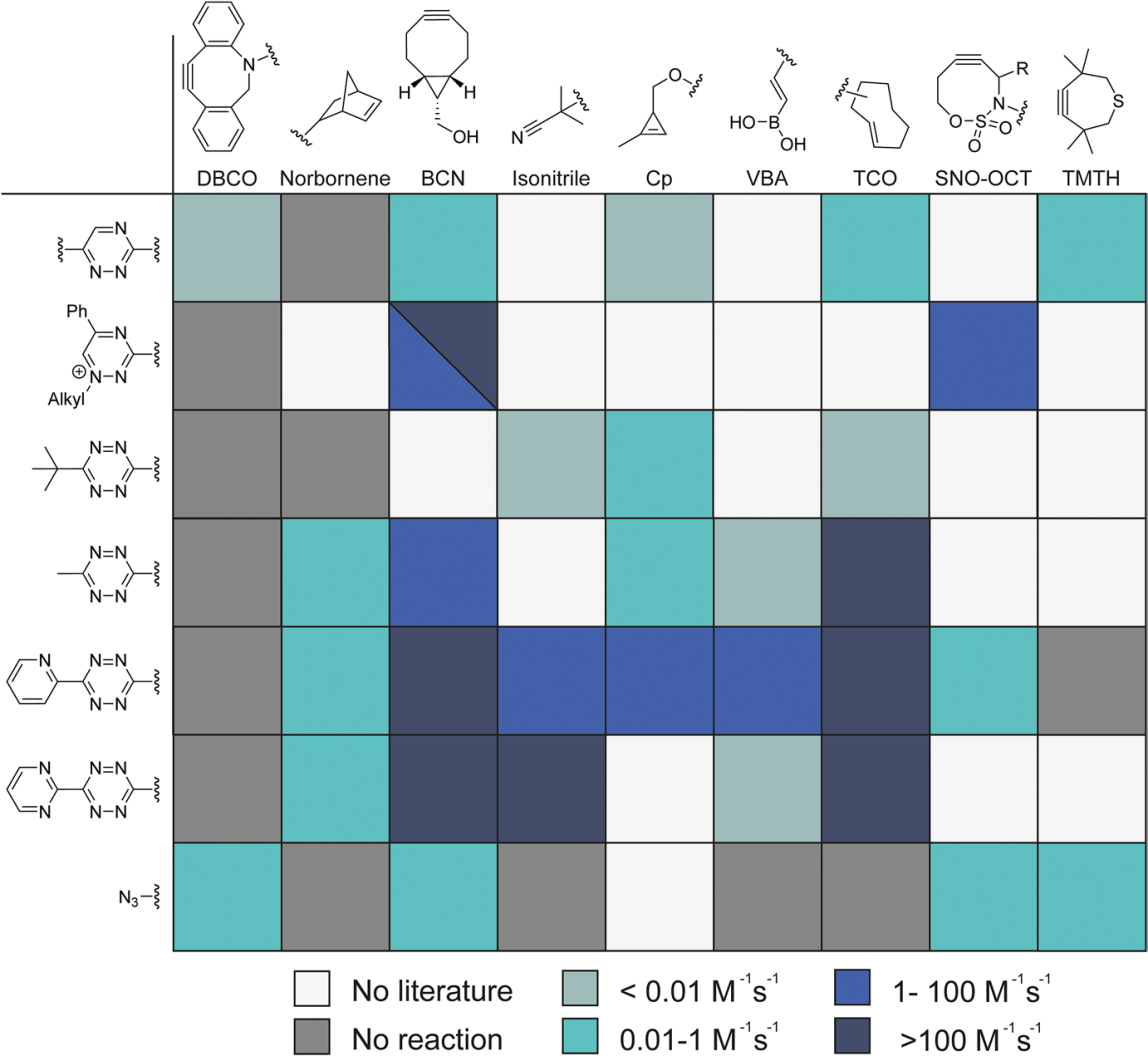
Matrix depicting reaction kinetics between several types of tetrazines and triazines with dienophiles [56, 60, 62, 64, 65]

Other dienophiles include cyclooctynes, such as BCN and DIFO, norbornenes, and methylcyclopropenes (MCps) [45, 46, 57]. More sterically hindered cyclooctynes, such as DBCO and TMTH, do not react with tetrazines [58–60]. A recent promising class of dienophiles are the S-, N-, and O-containing cyclooctenes (SNO-OCT). They benefit from their unique electronic tuning properties and a sulfonyl group, which increases their polarity and stability [61, 62]. SNO-OCTs react reasonably with dienes, azides, and diazo compounds and react slower with azides compared with BCN, providing the possibility for orthogonal use with the SPAAC reaction [61]. The reaction of SNO-OCT with azides, however, can be increased by two orders of magnitude by the introduction of electron withdrawing substituents on the SNO-OCTs [62]. Figure 5 outlines the relative kinetics of the IEDDA reactions and potential reactions with an azide. This also includes the vinylboronic acids (VBAs) [63] that uniquely react with high rates to tetrazines bearing a boron-coordinating substituent while no reactivity is observed to tetrazines with non-coordinating substituents, as additionally discussed in Sect. 3.4.2.

#### Tuning Reactivity in the Inverse Electron Demand Diels–Alder Cycloaddition (IEDDA) Reaction

**Fig. 6:**
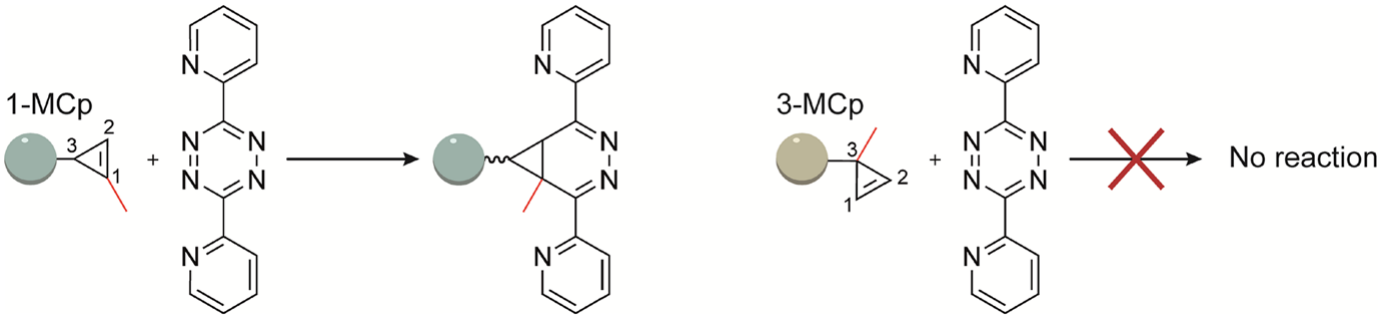
1- or 3-substitution on cyclopropenes strongly influences the capability to undergo click reactions with tetrazines [57]

The “distortion/interaction-activation strain model” helps to analyze how steric and electronic interactions influence the reactivity of dienophiles [44]. TCO derivatives have orders of magnitude decreased reactivity toward sterically encumbered tetrazines, whereas the much smaller 1,3-disubstituted cyclopropenes (Cps) are less sensitive to steric effects (Fig. 6) [57]. Within the Cps, the substituent pattern greatly affects reactivity toward tetrazines. Notably, the 1,3-disubstituted 1-methylcyclopropene (1-MCp) reacts orthogonally with tetrazines in the presence of 3-methylcyclopropene (3-MCp) [57].

**Fig. 7:**
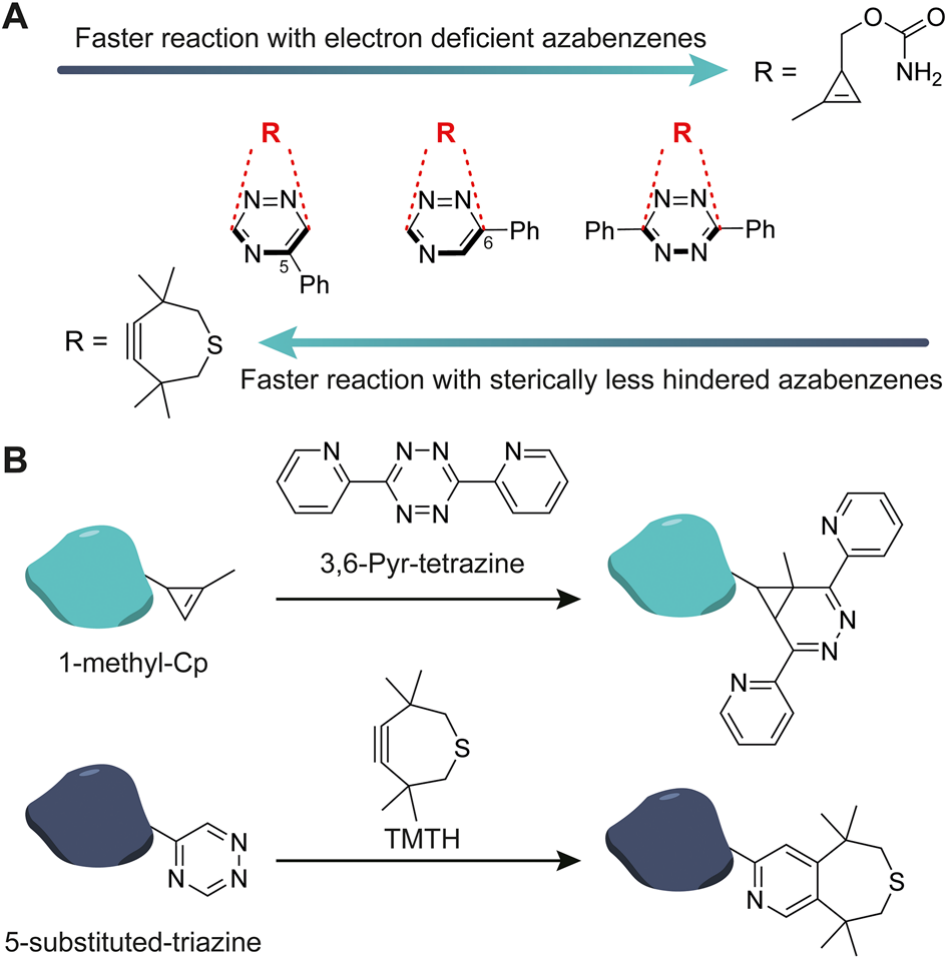
Development of mutually orthogonal reactions that arise from intrinsic reactivities and steric factors. **A** The reactivity trend of 5-phenyltriazine, 6-phenyltriazine, and 3,6-diphenyltetrazine with TMTH. 5-substituted triazines react with TMTH, even in the presence of tetrazine and Cp. **B** Dual orthogonal protein labeling through two IEDDA reactions by reacting Nluc-triazine and GFP-Cp with TMTH and 3,6-Pyr-tetrazine. The general approach for developing mutual orthogonal reaction by balancing electronic interactions (number of nitrogen atoms in the heterocycle) with steric effects [60]

The low reactivity of triazines prevents reactions with less reactive alkenes, such as norbornene or Cps, providing mutual orthogonality with tetrazines (Fig. 5 and 7) [54]. Therefore, the triazine-TCO pair can be combined with norbornene or Cp and a tetrazine in a sequential orthogonal strategy. Here, the less reactive norbornene or Cp first reacts with the tetrazine, after which a TCO is added to react with the remaining triazine.

Bulky dienophiles, such as TMTH, are unreactive to 3,6-substituted tetrazines and -triazines owing to sterical hindrance from its methyl groups. They do react, however, with 5-substituted 1,2,4-triazines [60]. Sterically less hindered 1-MCp has a higher reactivity toward more electrophilic dienes and therefore reacts faster with tetrazine and slower with the 5-substituted triazine. Prescher demonstrated the orthogonal use of 1,2,4-triazine and 1-MCp for labeling of two biomolecules simultaneously. In this study, a nanoluciferase and GFP protein that were labeled with a 1,2,4-triazine and 1-MCp reacted with TMTH and dipyridyl tetrazine, respectively (Fig. 7) [60]. As already highlighted previously, the TMTH compound is unstable and not suited for conjugation to a ligand. To our knowledge, the improved TMTH-SI has not been tested on triazines.

### Phosphine Ligations

**Fig. 8:**
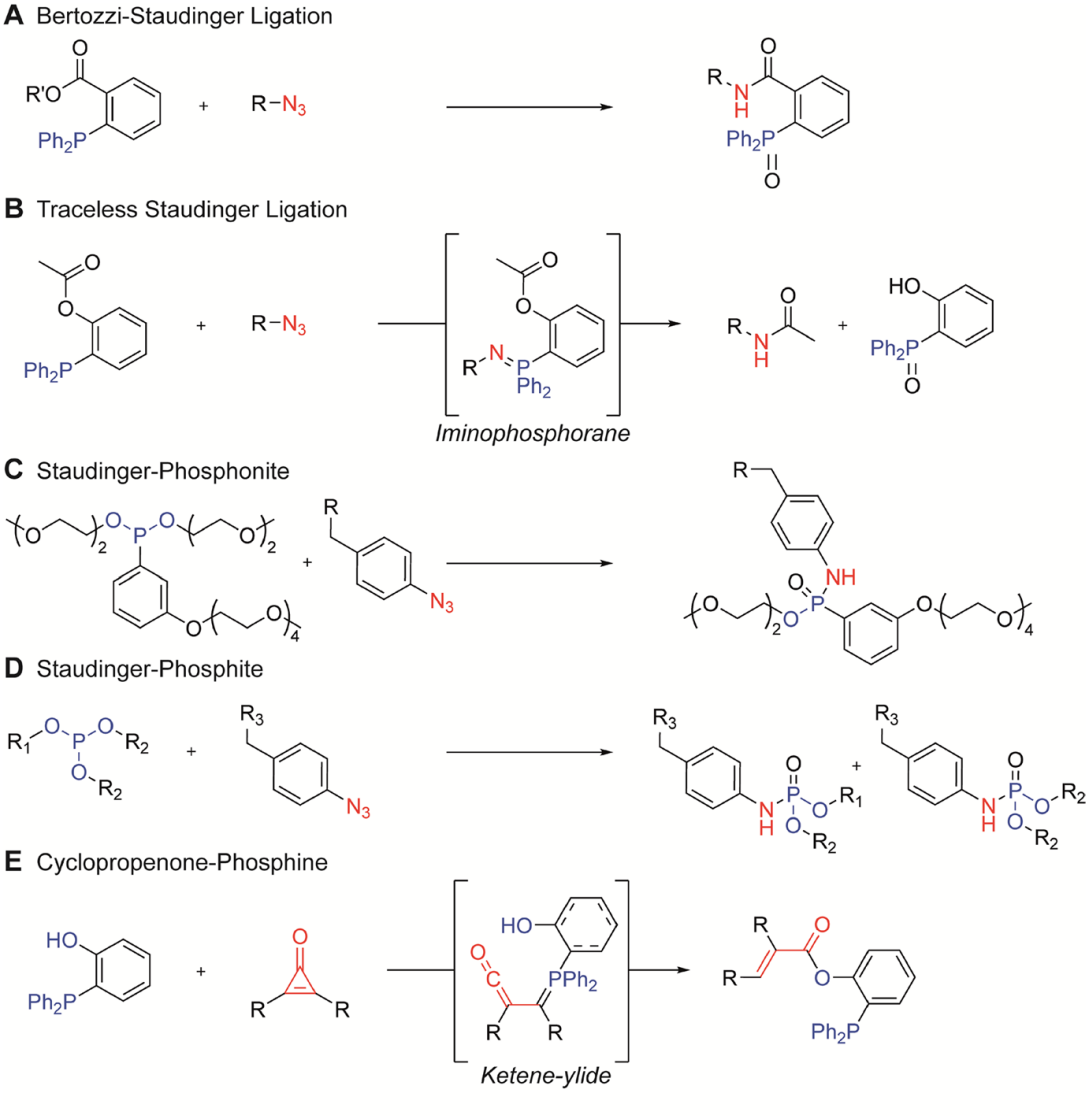
Overview of the **A** Bertozzi-Staudinger ligation, **B** “traceless” Staudinger ligation with the iminophosphorane intermediate, **C** the Staudinger-phosphonite, **D** Staudinger-phosphite, and **E** cyclopropenone-phosphine ligations

The Staudinger-Bertozzi ligation (SBL) involves the reaction between an organic azide and a modified triaryl phosphine, and is one of the first bioorthogonal reactions used for labeling of biomolecules in vivo [66]. In the classic Staudinger reaction, an azide reacts with the phosphine into an iminophosphorane that hydrolyses to an amine and phosphine oxide in aqueous conditions. In the SBL an electrophilic methyl ester is present on one of the phosphine aryl ligands that serves as an internal trap for the imino group and results in the formation of a covalent amide linkage (Fig. 8) [67]. An alternative reaction variant is the traceless Staudinger ligation introduced by Raines et al. [68]. The reaction between phosphane and azide is “traceless” as the phosphine scaffold is absent and reagents are connected by a single unsubstituted amide linkage [69]. Alternative phosphines include the Staudinger-phosphite reaction [70] and the Staudinger phosphonate (Fig. 8) [71]. The drawbacks of these phosphine-based ligations are the slow reaction rates and the sensitivity of the phosphine toward oxidative environments [72]. The phosphite has a higher resistance to oxidation compared with the trialkyl phosphines, but can hydrolyze at acidic or even neutral pH, depending on their substituents [73]. Phosphinothiol show improved reaction kinetics, but the increased electron density also leads to higher susceptibility to oxidation [74]. Borane-protected phosphinothioesters were found to be more stable toward oxidation [74]. Recently, Poulou & Hackenberger summarized phosphine mediated ligations in an extensive review on the development and applications of the Staudinger ligation in labeling of biomolecules [75].

The use of cyclopropenones (CpOs), developed by Prescher’s group, proved a viable alternative to the azide in phosphine ligations [76]. In here, CpO reacts efficiently with triphenylphosphine via a Michael-type addition to form a ketene-ylide intermediate (Fig. 8E) [76]. Not all CpOs are stable in an intracellular environment, but adding steric bulk improves their stability. For example, introducing dialkyl-substitutions around the electrophilic carbonyl carbon prevents undesired competing nucleophilic thiol attacks [77]. In addition, altering heteroatoms can affect the reaction rates. For example, cyclopropenethione (CpS) react with substituted phosphines to provide thiocarbonyl adducts, sometimes 300-fold faster than CpO [78]. Cyclopropeniminium (CpN^+^) motifs, with NR_2_ instead of the oxygen of CpOs, have a unique reactivity pattern and allow for some orthogonality with CpOs in phosphine ligations [79].

### [4 + 1] Cycloaddition with Isonitriles

**Fig. 9:**
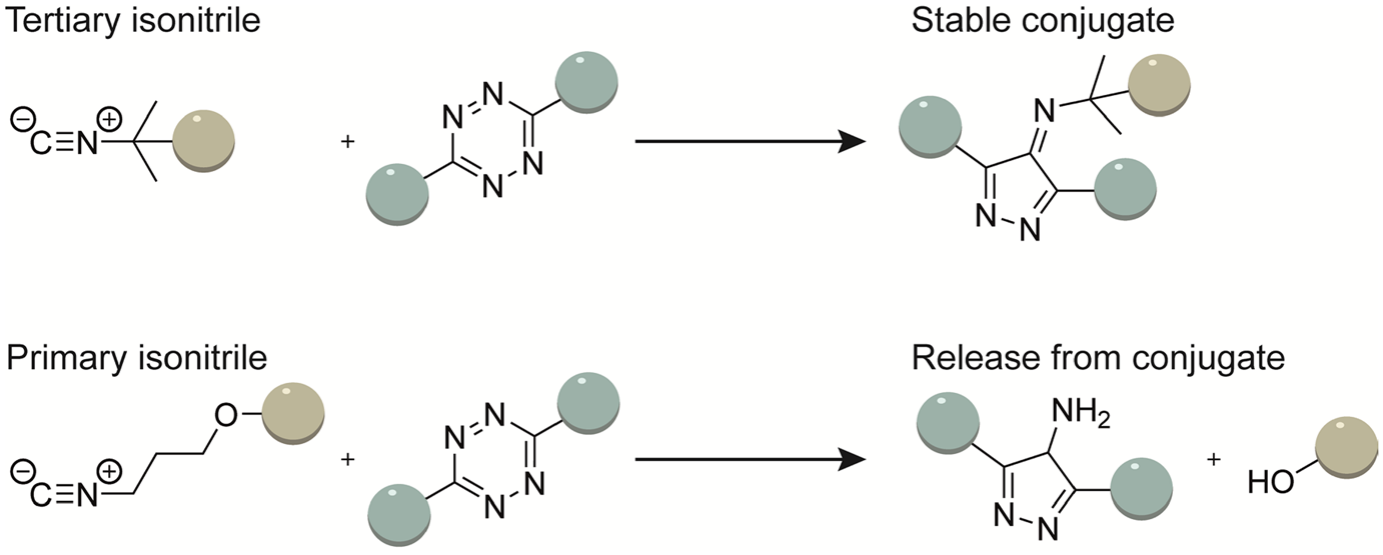
The reactions between tetrazines and tertiary and primary isonitriles create stable and unstable conjugates respectively

Isonitriles react in a [4 + 1] cycloaddition with tetrazines or chlorooximes (Fig. 1) [80, 81]. The reaction is followed by a [4 + 2] cycloreversion that liberates nitrogen while concurrently generating a novel pyrazole ring [80]. The use of tertiary isonitriles provide stable products in the isonitrile-tetrazine ligation, while primary isonitriles form labile pyrazole products that are prone to hydrolysis [80]. For this reason, Franzini explored the use of primary isonitriles as caging group that can be selectively released using a chemical tetrazine trigger (Fig. 9) [82].

Isonitriles are able to uniquely react with sterically encumbered 3,6-bis-tert-butyl tetrazine (DTTz), while strained alkenes/alkynes are inreactive to DTTz owing to the steric bulk [83]. The stability-enhancing bulky tetrazine substituents are rate-enhancing and primary and tertiary isonitriles can react four- to fivefold faster with bulky tetrazines compared with less bulky tetrazines [83]. DTTz are stable reactants as the bulky groups prevent more hindered dienophiles and nucleophile from approaching. The use of a sterically hindered tetrazine thereby allows for orthogonal use of the IEDDA reaction and the isonitrile [4 + 1] cycloaddition for sequential orthogonal labeling of biomolecules. Both the isonitrile-tetrazine and isonitrile-chlorooxime ligations are additionally orthogonal to the SPAAC reaction [81, 84].

### Condensation with Boronic Acids

Boronic acids are a distinctive class of compounds with unique electronic properties attributed to their vacant p-orbital [63]. The difference in electronegativity between boron and carbon causes an inductive effect. The main exploited strategy is boronic ester condensation and imino boronate formation between 2-acetyl/formyl-aryl boronic acids and hydrazine/hydroxylamine/semicarbazide derivatives and used for many bioorthogonal ligations [85, 86]. The acylboronic acid-hydrazine ligation is considered bioorthogonal with certain hydrazine derivatives and allows for the irreversible formation of a boron-nitrogen heterocycle (diazaborine) derivative. Bane et al. introduced the 2-formylphenylboronic acid (2fPBA)-α-amino-hydrazide reaction, which forms a stable 2,3,1-benzodiazoborine derivative. The reaction is highly chemoselective and, more importantly, mutually orthogonal to the SPAAC and IEDDA reaction [87].

### Inducible Bioorthogonal Chemistry

The ability to obtain bioorthogonal reactants in an inducible and controlled manner using an external trigger allows for a high level of selectivity and extended spatial and temporal control in biological environments. In addition, the requirement of an external trigger adds an additional layer of reaction orthogonality. The development of (photo)chemically triggered click reactions has provided many new mutual orthogonal reaction partners to the most commonly used ones: CuAAC, SPAAC, and IEDDA.

#### Strain-Promoted Oxidation-Controlled Ortho-Quinone (SPOCQ) Cycloaddition

The strain-promoted oxidation-controlled cyclooctyne–1,2-quinone cycloaddition (SPOCQ) proceeds several orders of magnitude faster with strained alkynes and alkenes when compared with azides as explored by van Delft [88]. The 1,2-quinones are formed from catechols using mild oxidation conditions (e.g. sodium periodate) or from tyrosines using tyrosine peroxidase enzyme [89]. The quinones react uniquely fast with BCN when compared with TCO and TMTH(-SI) and showed very low reactivity to DBCO [90]. The preferred reaction of 1,2-quinones with BCN allowed the orthogonal labeling of a BCN-modified protein in the presence of azides [88].

#### Enzymatic Activation of Caged Tetrazines

Knittel et al. prepared a dihydrotetrazine conjugated with an enzyme cleavable group and several different self-immolative linkers [91]. Enzymatic cleavage leads to release of the dihydrotetrazine that spontaneously oxidizes to a functional tetrazine [91]. The authors generated caged tetrazines for enzymes that are overexpressed in certain cancer cells. Using this strategy, the authors could induce an iEDDA click-to-release reaction of a dienophile-caged doxorubicin drug in cells that express high levels of enzyme.

#### Photo-Click Cycloadditions

**Fig. 10:**
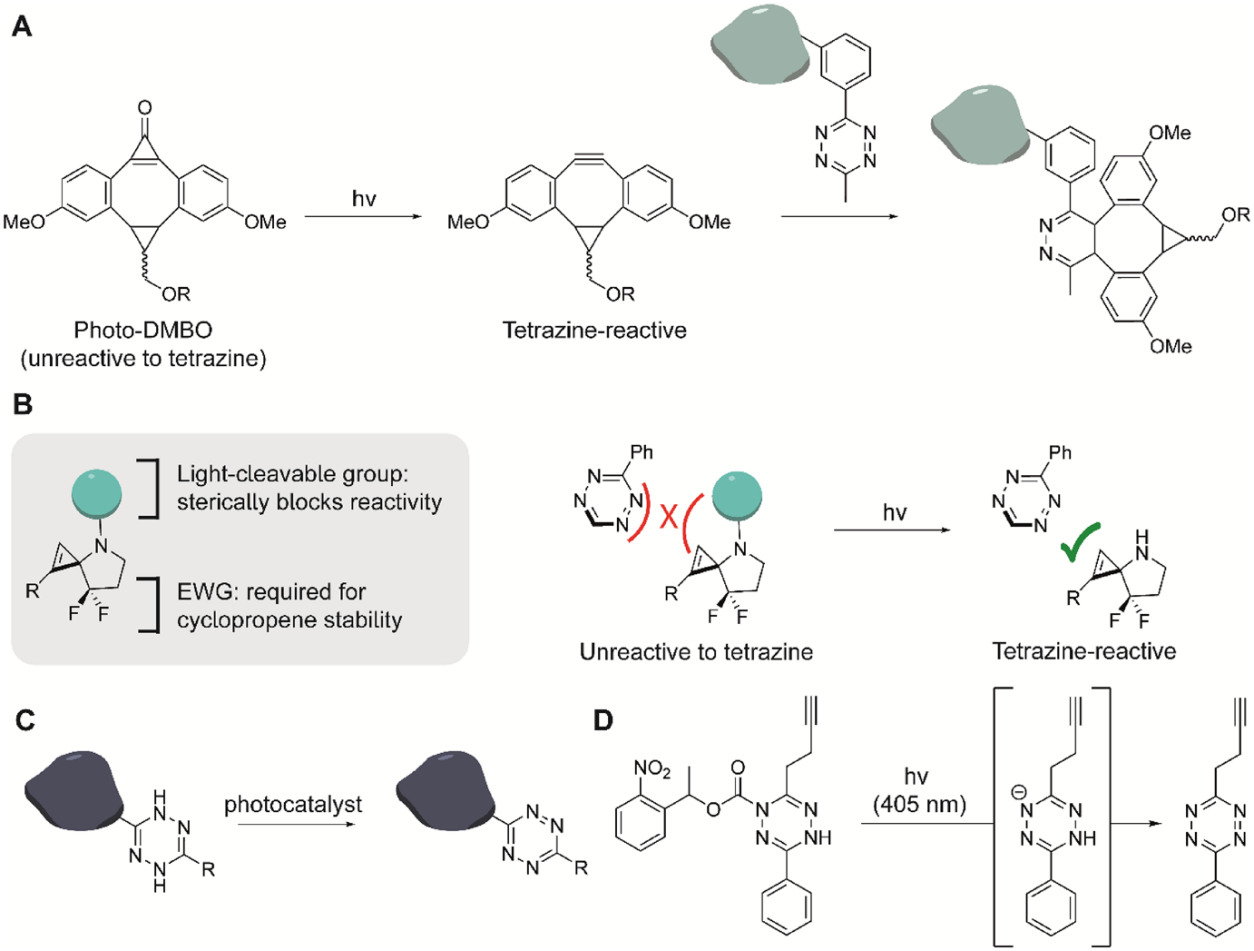
Photocaged click reagents. **A** CpO-caged bicyclononynes are light-induced for tetrazine ligation using visible light [95]. **B** Modular caging strategy with bulky Cp “reactivity cages’’ where attachment of photolabile groups at the cyclopropene nitrogen prevents ligation [97]. **C** Tetrazine oxidation via excitation of a photocatalyst. **D**) Photodecaging of a protected tetrazine at 405 nm

Photocaged compounds that become active after irradiation can also be used for sequential mutual orthogonal strategies [92, 93]. An interesting example is the photo-induced activation of CpO-caged cyclooctynes, which can be used for photo-SPAAC and photo-IEDDA to create mutual orthogonal reactions [94]. Here, Lang reported on a CpO-caged dibenzoannulated BCN derivative (photo-DMBO), a photo-activatable BCN-based probe. Ultraviolet (UV) irradiation at 365 nm decarbonylates the compound to DMBO (Fig. 10A) [95]. A site-specific incorporation of lysine-based methyl-substituted tetrazine of amino acids in *Escherichia coli* illustrates the mutual orthogonality of photo-DMBO labeling and IEDDA reactions with tetrazines. This strategy was also used with 3-*N*-substituted spirocyclopropenes, a photocaged Cp [96, 97]. The bulky *N*-protecting groups sterically prohibit reaction with tetrazines owing to steric repulsion. Upon exposure to light, the Cp is decaged, removing the steric block and allowing the reaction with the respective tetrazine (Fig. 10B).

The group of Fox developed a method to form tetrazines in situ from inactive dihydrotetrazine precursors using a suitable photocatalyst and light [98]. Incubation with silarhodamine or fluorescein dyes and irradiation at 660 nm or 470 nm, respectively, oxidizes the dihydrotetrazine into a functional tetrazine that can undergo IEDDA cycloaddition with strained dienophiles (Fig. 10C). This spatial addition of light allows tight spatial and temporal control of protein labeling in living cells [98]. Alternatively, Devaraj and coworkers modified the secondary amine in the dihydrotetrazine precursor with a photoprotecting group, which removes the need for a photocatalyst. Using a nitrophenyl deriverative as photocage, irradiation at 405 nm removed the photoprotecting group, leading to subsequent oxidation of the dihydrotetrazine into a functional tetrazine (Fig. 10D) [99].

**Fig. 11:**
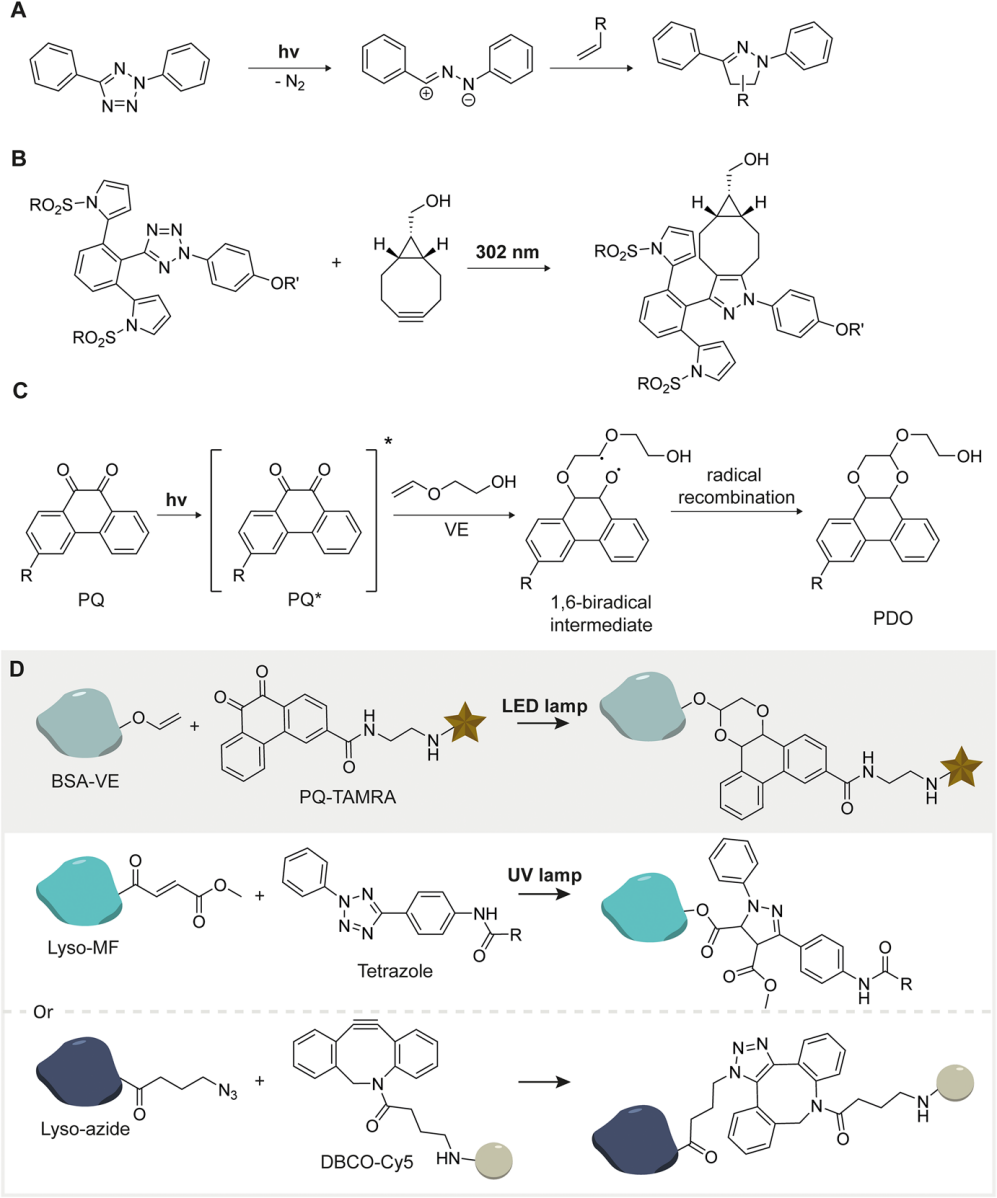
Mutual orthogonal labeling with visible light initiated photoclick cycloaddition. **A** UV-induced reaction of 2,5-diphenyl tetrazoles with alkenes. **B** Sterically shielded sulfonated tetrazoles allows selective reaction with BCN. **C** Visible-light-induced [4 + 2] cycloaddition between PQ and VE, where PQ is exposed to visible light, becomes excited (PQ*), and transfers electrons to VE through photoinduced electron transfer (PeT). This electron transfer leads to the formation of a highly reactive 1,6-biradical intermediate. The intermediate then undergoes intramolecular radical recombination, forming [4 + 2] cycloadduct with the phenanthrodioxine (PDO) framework [104]. **D** Two mutual orthogonal labeling reactions of BSA-VE with either LYSO-MF or LYSO-azide. The first orthogonal fluorescent labeling was achieved between reaction pairs BSA-VE and LYSO-MF through the reaction with PQ-TAMRA and tetrazole, respectively (d, top)—the second reaction of BSA-VE and LYSO-azide through the reaction with PQ-TAMRA and DBCO-Cy5 (d, bottom) [103]

Tetrazoles form reactive nitrile imines upon exposure of UV light. The Lin’s group developed tetrazole-ene photo-click chemistry (TPEC), where UV light triggers the generation of nitrile imines from 2,5-biaryl tetrazoles that subsequently react with alkenes (Fig. 11A) [100, 101]. Recently, Lin and coworkers introduced a new class of sterically shielded, sulfonated tetrazoles. The reagents react extremely fast and preferentially with BCN over strained alkenes and provide one of the fastest bioorthogonal ligations with BCN to date (Fig. 11B) [102].

The vinyl ether photo-cycloaddition (DVPC) reaction developed by Zhang et al. is a visible-light-induced [4 + 2] cycloaddition between 9,10-phenanthrenequinone (PQ) and vinyl ethers (VE) that occurs under biocompatible conditions (Fig. 11C) [103]. The reagents are unreactive toward strained alkynes, electron-deficient alkenes (such as monomethyl fumarate, **MF**), and other nucleophilic species. The authors observed some reactivity with TCO but at lower rates compared to VE. This allows mutual orthogonality with the azide-DBCO and UV-initiated tetrazole-ene photoclick reaction (Fig. 11D) [103].

#### Wavelength Selectivity and Photocaging for Orthogonality

**Fig. 12:**
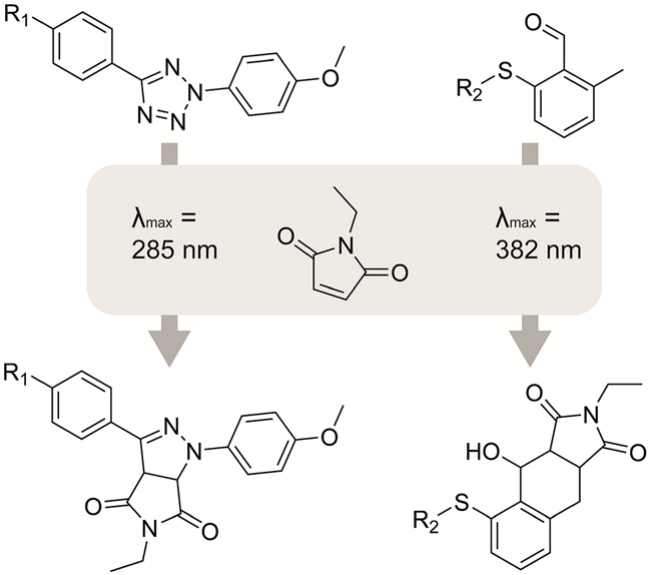
Selective photoclick reactions based on the wavelength. At 285 nm 2,5-diphenyl tetrazoles can react with N-ethylmaleimide to form the pyrazoline ligation product, while at 382 nm o-methyl benzaldehyde can react with N-ethylmaleimide

Orthogonality in photoclick reactions can be achieved by using different wavelengths, in so-called *λ*-orthogonality or wavelength selectivity [105]. Kowollik et al. reported a light-induced reaction in a dual-chromophore setup where reactivity depended solely on the choice of wavelength and solvent. Here, the conversion of 2,5-diphenyl tetrazoles with N-ethylmaleimide at *λ*_max_ = 285 nm yielded pyrazoline ligation products. Simultaneously, at *λ*_max_ = 382 nm, o-methyl benzaldehyde reacted with N-ethylmaleimide via an o-quinodimethane intermediate while retaining the 2,5-diphenyl tetrazoles (Fig. 12) [106].

## Tuning the Reaction Partners for Dual and Triple Orthogonal Bioorthogonal Labeling

### Combining Mechanistically Different Bioorthogonal Reactions

**Fig. 13:**
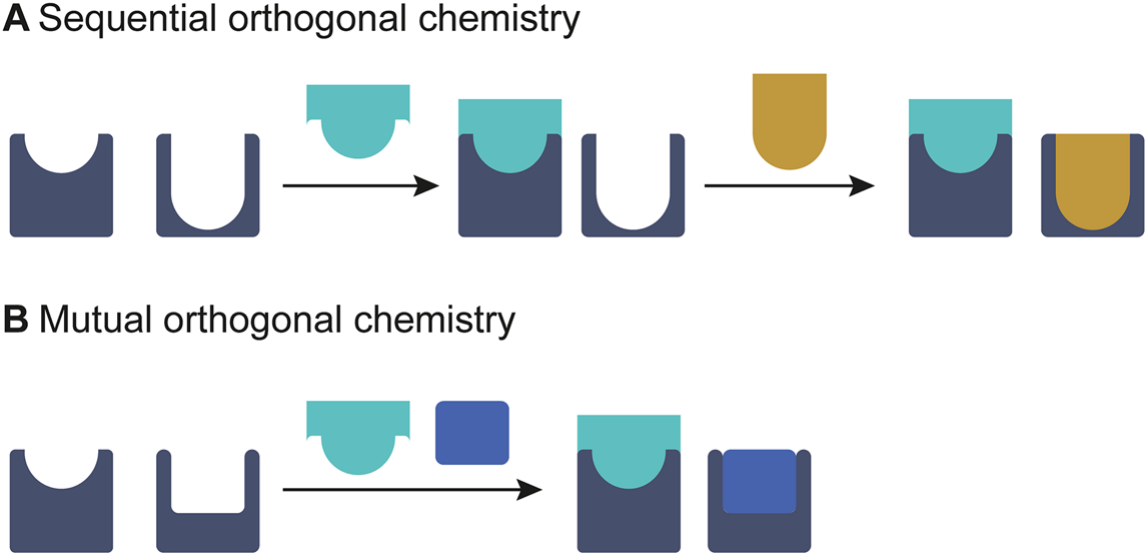
**A** Sequential orthogonal click chemistry in which order of addition or activation of reactants is crucial. **B** Mutual orthogonal approach in which all reactants are present simultaneously

Site-selective dual functionalization of proteins using mutual orthogonal chemistry reactions has been reported in various approaches. In this context, it is important to note that next to mutual orthogonal chemistry where all reagents are added simultaneously in a single pot, there are many strategies that require the sequential addition of reagents, leading to a sequential orthogonal labeling approach (Fig. 13). Weil et al. and Chen & Wu summarized the bioorthogonal chemistry combinations generally employed in literature for single or dual protein modification [107, 108]. The approaches included are (I) CuAAc + SPAAC (sequential orthogonal) [109], (II) IEDDA + IEDDA (sequential orthogonal) [110], (III) SPAAC + IEDDA (sequential or mutual orthogonal) [111], (IV) CuAAC + oxime ligation (mutual orthogonal) [112], and (V) CuAAC + IEDDA (mututal orthogonal) [113].

The tetrazine-TCO IEDDA and DBCO-azide SPAAC reactions are often used in combination for dual modification of biomolecules as they are mutual orthogonal and many variants of the reagents are commercially available. For example, Alabi’s group used the IEDDA and SPAAC combination for dual modification of an antibody to obtain bifunctional antibody–drug conjugates (ADCs) [114]. In here, the authors chemically engineered the Her2-targeted antibody trastuzumab with a linker containing a methyltetrazine as well as an azide handle. The engineered antibodies were consequently functionalized with DM1 toxin as well as a hydrophylic PEG linker in a dual click reaction using TCO-DM1 and DBCO-PEG that react with the tetrazine and the azide, respectively.

**Fig. 14:**
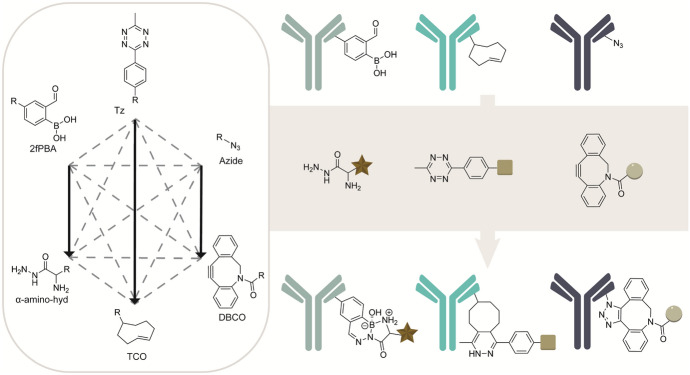
Mutual orthogonal triple labeling of antibody–drug conjugates by adopting three reaction pairs proceeding through distinct mechanistic pathways. Three trastuzumab antibodies functionalized with either 2-fPBA, azide, or TCO reacted with fluorophores containing the complementary functional groups, α-amino-hydrazide-RED, DBCO-TAMRA, and Tetrazine-BODIPY. The mutual orthogonality of the boronic acid condensation, SPAAC, and IEDDA reactions enable simultaneous triple labeling [87]

The IEDDA and SPAAC reactions can be further expanded using other reactions that are mechanistically different, such as the bioorthogonal boronic acid condensation between 2fPBA and α-amino-hydrazide. For example, bone functionalized trastuzumab antibodies with either 2fPBA, azide, or TCO. Multi-bioorthogonal labeling with α-amino-hydrazide-RED, DBCO-TAMRA, and Tetrazine-BODIPY, respectively, resulted in the simultaneous triple antibody labeling on the basis of three distinct mechanisms (Fig. 14) [87].

**Fig. 15:**
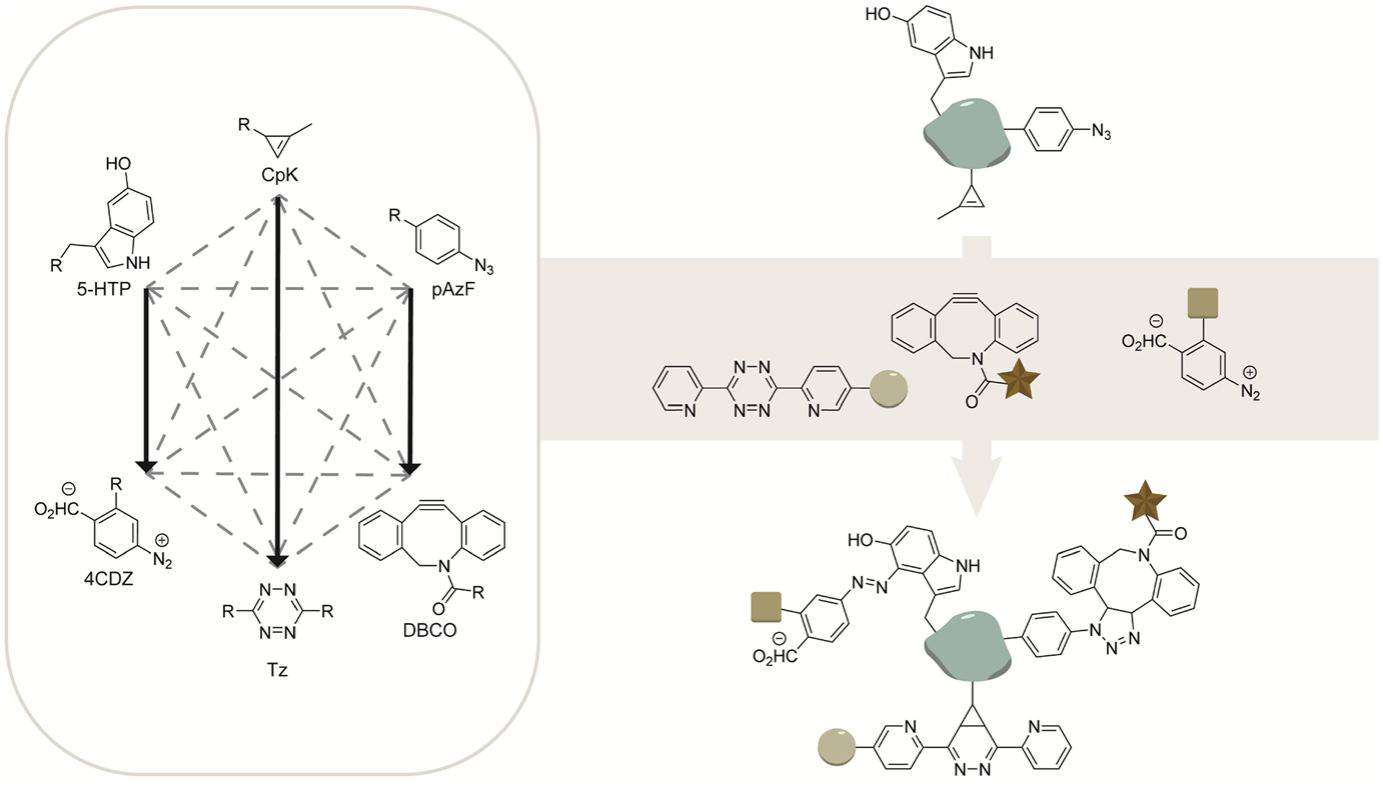
CRACR-mediated labeling of 5-HTP, SPAAC-labeling of pAzF, and IEDDA labeling of CpK, achieving mutual orthogonal triple labeling in proteins [115]

Chatterjee et al. expanded the combination of SPAAC and IEDDA reactions with a chemoselective rapid azo-coupling reaction (CRACR) for site-specific bioconjugation and applied this for site-selective labeling in proteins. In here, 5-hydroxytryptophan (5HTP) residues are labeled chemoselectively with aromatic diazonium ions using the high electron-rich 5-hydroxy indole group. Using genetic code expansion, three non-canonical amino acids containing different bioorthogonal functional handles, e.g., 5-HTP, pAzF, and CpK (Fig. 15) were site selectively introduced into a protein [115]. The incorporated bioorthogonal groups were functionalized using CRACR-mediated labeling of 5-HTP with diazonium ion, SPAAC-labeling of pAzF with DBCO, and IEDDA labeling of CpK with a tetrazine, achieving protein labeling at three distinct sites [115]. This study shows that the CRACR is compatible with the SPAAC and IEDDA reactions for triple mutual orthogonal labeling [116].

### Electronic Tuning of Reaction Partners

**Fig. 16:**
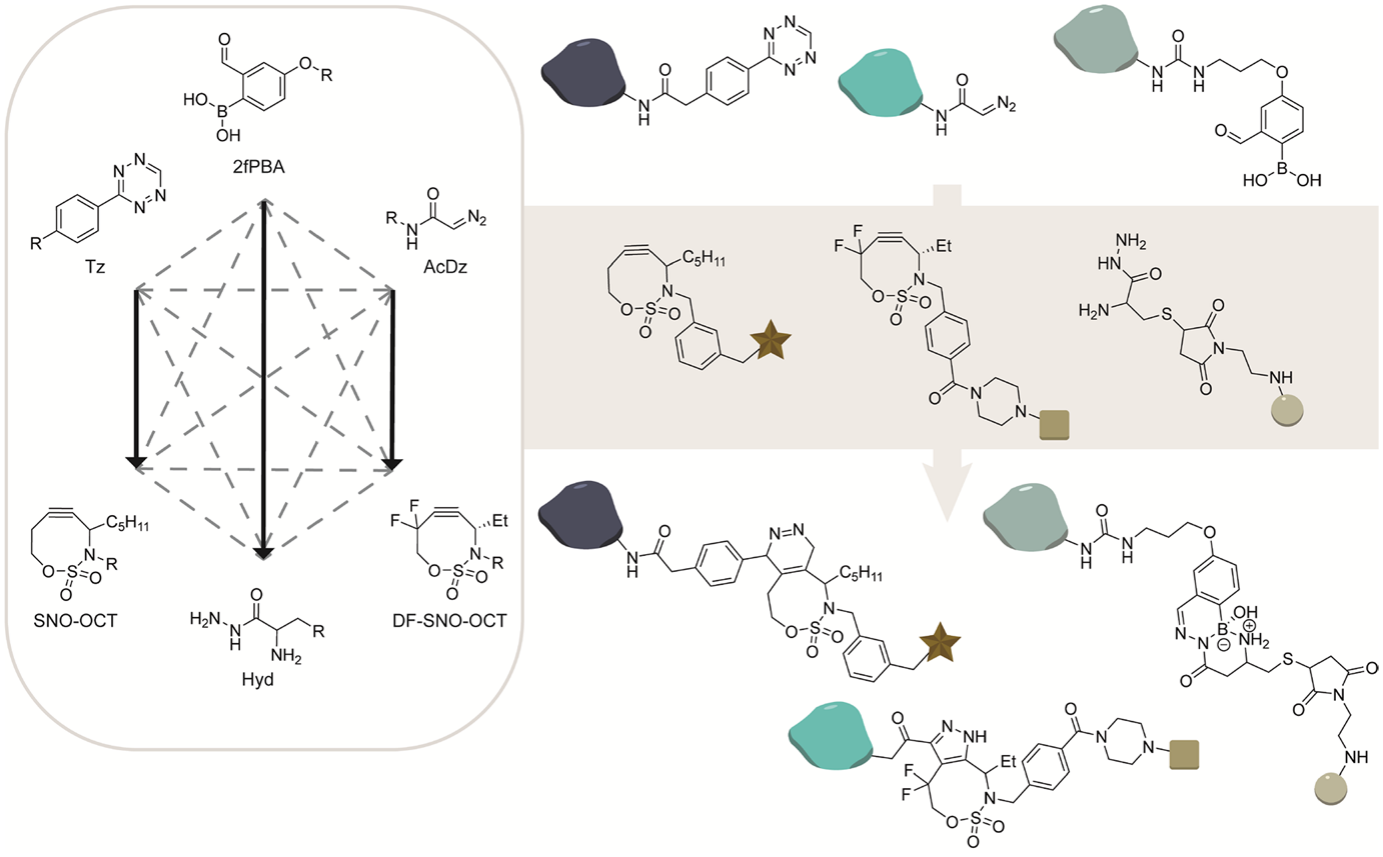
Mutual orthogonal triple labeling of proteins through electronic tuning of the alkyne. The SNO-OCT derivatives were designed with substitution patterns to tune the alkyne electronics tailored toward type I and III dipoles. The boronic acid condensation with α-amino hydrazides, which is mechanistically different, was included as a third mutual orthogonal reaction [62]

Mutual orthogonal click reactions within the same class of reactants can be achieved via electronic tuning of reagents. Raines’ and Schomaker’s groups showed that by tuning the electronic properties of SNO-OCTs, they could direct the preference for particular type I or III dipoles [62]. In this study, three proteins were individually functionalized with a diazoacetamide dipole (Cytoc-AcDz), a tetrazine dipole (RNase A-tet), and boronic-acid–aldehyde (RNase A-BA) and reacted with three fluorophores containing the respective complementary bioorthogonal handles: difuoro-substituted DF-SNO-OCT-Rho, parent P-SNO-OCT-C343, and alpha-amino-hydrazide-T (Fig. 16). Triple labeling was achieved simultaneously with SPAAC, IEDDA, and the boronic acid condensation, demonstrating the mutual orthogonality of the three reaction pairs [62].

### Steric Tuning of Reaction Partners

**Fig. 17:**
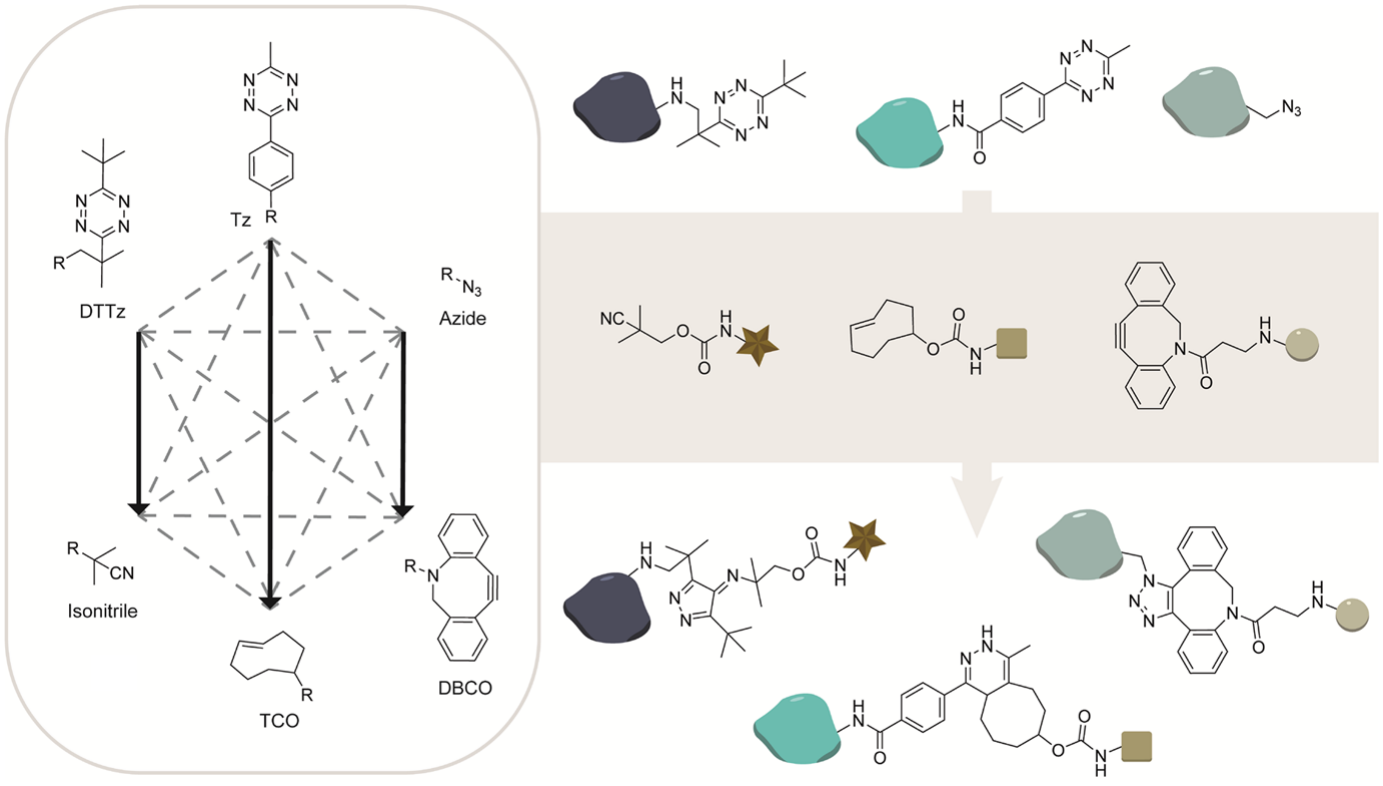
One-pot strategy with triple mutual orthogonal labeling based on dispersion forces between the isocyano group and tetrazine substituents. Three proteins, ovalbumin (OVA), bovine serum albumin (BSA), and lysozyme (LYSO), were modified with bioorthogonal handles azide, 3,6-bis-tert-butyl tetrazine (DTTz), and MeTzCOOH, respectively. The functionalized proteins were reacted with three fluorophores with complementary reactive groups, DBCO-AF405, TCO-BODIPY red, and tertiary isonitrile-BODIPY green [83]

Steric tuning of the reactants is an alternative strategy to obtain mutual orthogonal reaction pairs. For example, isonitriles uniquely react with bulky tetrazines, while strained alkynes/alkenes are unreactive and preferentially react with tetrazines containing small substituents. Building on this fact, Franzini’s group modified ovalbumin (OVA), bovine serum albumin (BSA), and lysozyme (LYSO) with an azide, a bulky 3,6-di-tert-butyl tetrazine (DTTz) and less hindered methyl phenyl tetrazine, respectively (Fig. 17) [83]. A selective reaction of DTTz with a tertiairy isonitrile-BODIPY green and methyl phenyl tetrazine with TCO-BODIPY-red was indeed feasible. The mechanistically different SPAAC reaction with DBCO-AF405 was used as the third mutual orthogonal reaction. Kele et al. used a similar approach to induce tetrazine orthogonality. In this dual labeling example, cells using genetically encoded nonnatural amino acids containing a BCN and a bulky isonitrille was used to react selectively with a methyl phenyl tetrazine instead and a bulky tetrazine, respectively [117].

**Fig. 18:**
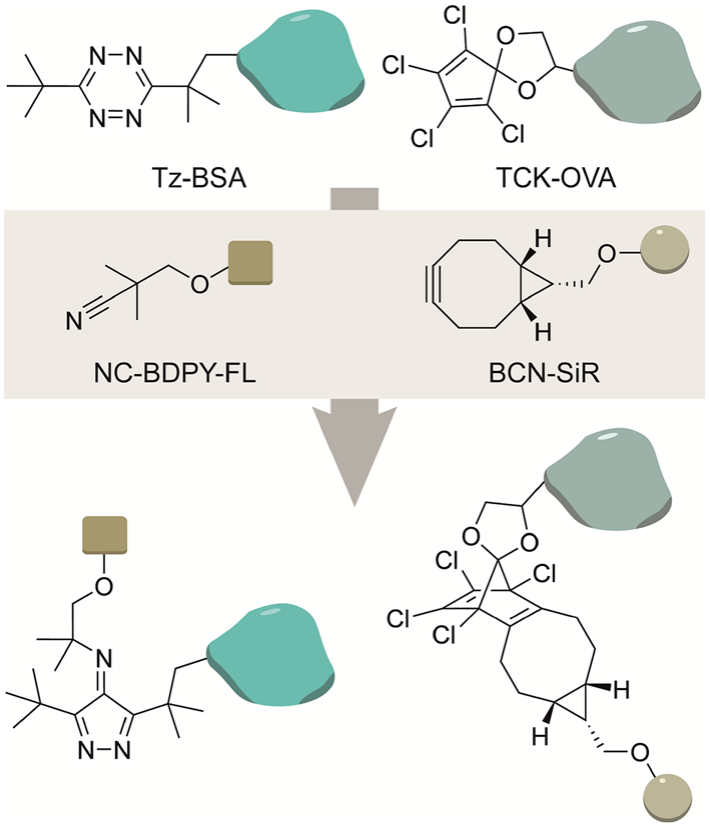
Mutual orthogonal chemistry between tert-butyl tetrazine conjugated to BSA and isonitrile-bodipy and tetrachlorocyclopentadienone ethylene ketal conjugated to OVA and BCN-SiR

Franzini and coworkers tested chemoselective IEDDA reactions on the basis of different dienes. They established that five-membered cyclic dienes (FMCDs) are inert to isonitriles but readily react with strained alkynes. Consistent with their previous findings that tetrazines with bulky substituents readily react with isonitriles, they performed a dual orthogonal protein-labeling experiment. Modifying two proteins with either a bulky DTTz or tetrachlorocyclopentadienone ethylene ketal (TCK) FMCD allowed selective protein modification using an isonitrile and a BCN, respectively (Fig. 18) [118]. In this example, the fluorophore was conjugated to the isonitrile. Tetrazines conjugated to fluorophore scaffolds allow for tunable “turn-on” fluorescent probes once reacted with a bulky isonitrile [119]. Alternatively, tert-butyl tetrazine caged fluorophores can be released upon reaction with a (bulky) isonitrile [120].

### Inducing Reactant Proximity

#### Coordination Assisted Bioorthogonal Chemistry

**Fig. 19:**
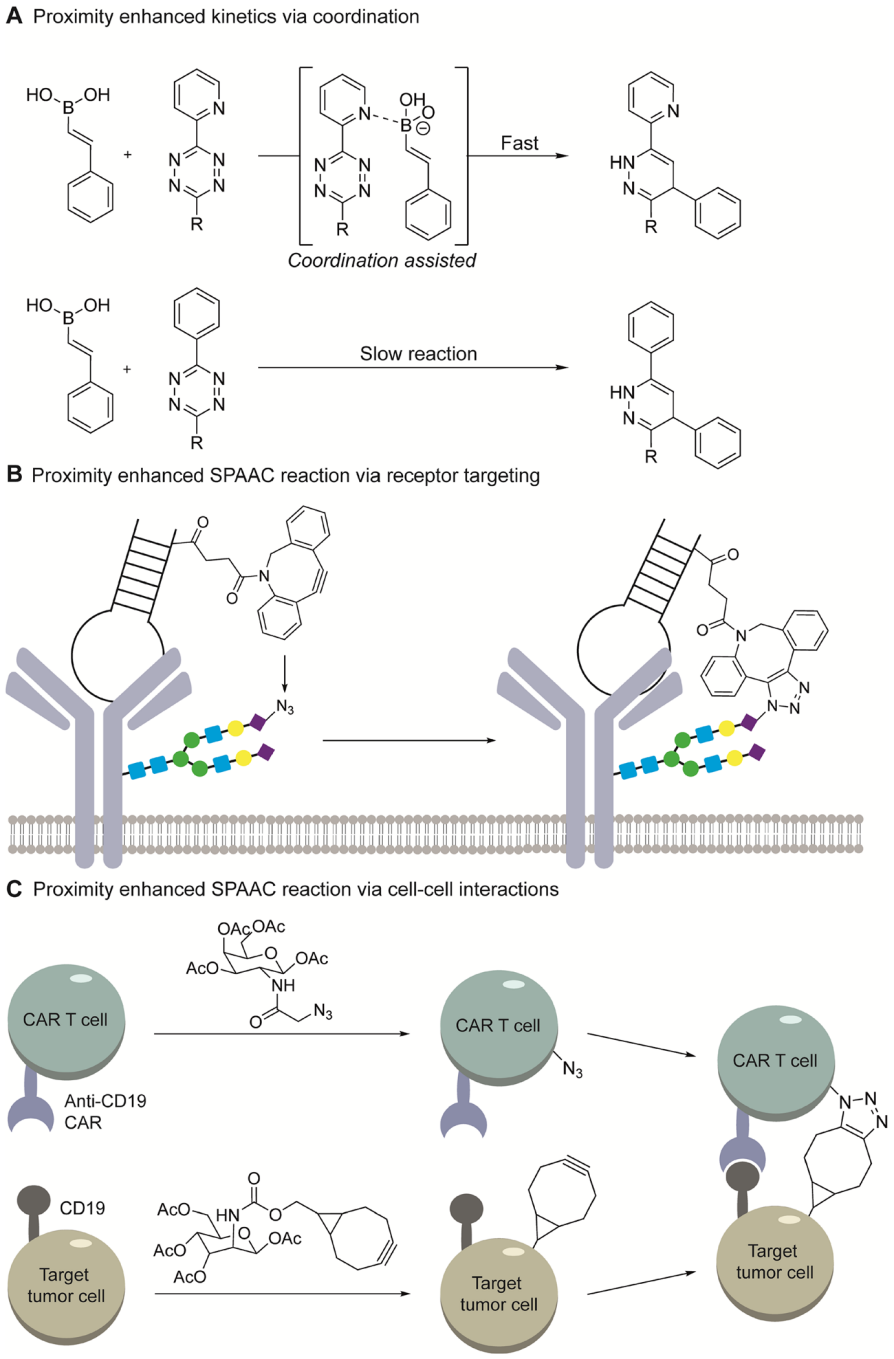
Proximity enhanced click chemistry. **A** VBA uniquely react with tetrazines containing substituents that coordinate the boron atom. **B** An aptamer conjugate specifically binds to the target receptor and brings the DBCO in close proximity of an azide. **C** anti-CD19 CAR T cells can bind their target CD19 expressing cells, thereby bringing their metabolically labelled glycoproteins in close proximity, facilitating the click reaction

While it is possible to use non-strained alkenes with tetrazines for bioconjugations, the reaction is extremely slow as the rate constants is orders of magnitude below 1 M^−1^ s^−1^ and requires long reaction times to complete. Bringing the reactants in close proximity can accelerate the reaction rates several orders of magnitude. An example here is the use of vinylboronic acids (VBAs) that react with tetrazines via the IEDDA mechanism [63]. Fast reaction rates are observed when reacting VBA with tetrazines containing substituent that can coordinate to the boron atom, such as a pyridyl or an o-phenol, while VBAs are unreactive to tetrazines lacking such substituent (Fig. 19A) [121]. This coordination-assisted ligation can increase the reaction rate by several orders of magnitude making VBA suitable for a sequential two-step orthogonal ligation approach. In here, the VBA reacts first with a tetrazine containing boron-coordinating substituent, after which a strained alkene is added to react with a non-coordinating tetrazine [121].

#### Enhanced Kinetics Induced by Biomolecular Interactions

Researchers have also benefited from slow bioorthogonal reaction rates and used proximity-enhanced click chemistry to profile specific biomolecular interactions. This concept, introduced by Robinson, induces a high local concentration of the reactants to overcome their kinetic barrier [122]. In this study, they metabolically labeled cells with azido-modified sialic acid (SiaNAz) in surface glycoproteins [122]. Subsequently, they added a DBCO-modified aptamer that recognized either protein tyrosine kinase-7 or the mu heavy chain of the B cell receptor, which are expressed on Jurkat and Ramos cells, respectively [122]. Each DBCO aptamer only reacted with the SiaNAz when combined with its corresponding target receptor owing to the close proximity, whereas no reaction was observed with a non-specific DBCO-aptamer (Fig. 19B) [122]. These experiments highlight the potential for proximity induced rate enhancement due to a high local concentration of the click reagents, and makes use of slow bioorthogonal reaction pairs a viable option [122].

In another study, Pan’s group explored the effect of enhanced cell–cell interactions in chimeric antigen receptor (CAR) T cell cytotoxicity. Here, the researchers prepared anti-CD19 CAR T cells that were metabolically labeled with an azido-modified galactose analog on the cell surface [123]. The CD19 expressing target cells were treated with a BCN-modified mannose analogue (Ac_4_ManNBCN). Once the anti-CD19 CAR T cell and CD19 expressing target cell recognize each other, the reactants are in close proximity, allowing the azide-BCN SPAAC reaction to proceed (Fig. 19C) [123]. These examples highlight that slow kinetics can also be favorable when investigating cellular processes and can be used as an additional dimension to induce an orthogonal reaction pair.

## Considerations and Challenges in Mutual Bioorthogonal Chemistry

Bioorthogonal chemistry has made significant advancements over the last decades and many new reagents and reactions have been successfully explored for bioconjugations. In the context of mutually orthogonal bioorthogonal chemistry, the development of new reactions is continuously desired to improve and fine-tune reaction selectivity, reactant stability and ability to perform reactions in vivo.

Choosing the proper click chemistry pair highly depends on application, required conditions and (commercial) availability of the reagents. The most straightforward approach to achieve mutual orthogonality is by using two bioorthogonal reactions that proceed through different mechanistic pathways, such as the SPAAC and the IEDDA. Yet, within a mechanistic similar reaction pair it is possible to induce orthogonality by altering the steric and electronic properties of the reactants. Other strategies, such as activation triggers or to induce proximity of the reactants are possible to add another layer of control.

Additional desired features may also play a role. For example, the scope of click to release reactions is constantly expanding, with molecules that can release cargo or a quencher of their fluorescent moiety upon reaction completion [124]. So far, click to release has been reported from the Staudinger ligation [125], tetrazines [126], TCOs [127, 128], VBAs [129], iminosydnones [39, 40], and primary isonitriles [130]. Alternatively, there have been many examples for click to fluoresce, in which the click reaction yields a fluorescent dye or releases the quencher molecule to restore fluorescence [131–135]. This turn-on fluorescence is especially useful for imaging in living cells as no washing step due to excess fluorophore is needed.

### Reaction Kinetics put in Perspective

**Fig. 20:**
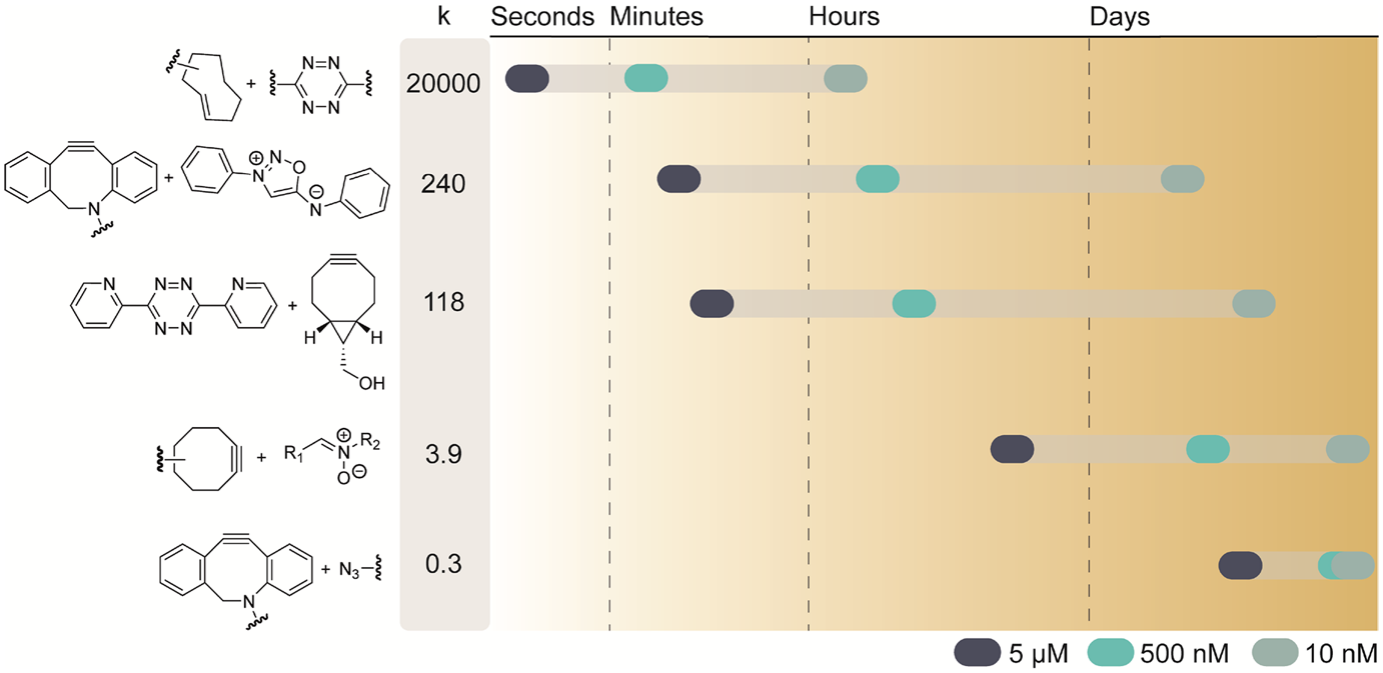
The estimated time needed to reach t_1/2_ of the reaction under ideal conditions, at 5 µM, 500 nM, and 10 nM. The reaction rates (K in Mol^−1^ s^−1^) for TCO-Tetrazine (~ 20,000) [48], DBCO-Iminosydnone [40], BCN-dipyridyl tetrazine [50], strained alkyne-nitrone [29], and DBCO-azide [21] click reactions were adapted from literature

When employing mutual orthogonal chemistry, the slowest pair will dictate the overall duration of the reaction. In general, a higher concentration or longer time for conversion is not as much a concern in making bioconjugates in situ as it is in live cell experiments. Using slow proceeding click reactions with rates < 1 M^−1^ s^−1^, in experimental settings that require low reactant concentrations, completion of the click reaction will not occur at a reasonable timeframe (Fig. 20). For example, a reaction with 100 µM of DBCO and azide, reacting with at *k*_2_ of 0.33 M^−1^ s^−1^, will take about 8.4 h to reach 50% conversion (*t*_1/2_), while at 5 µM it will take 7 days to achieve *t*_1/2_ [21]. However, any of these reactions can be performed in diluted conditions when the reactants are brought in close proximity to react with sufficient speed as described in Sect. 3.4.2.

### Stability and Toxicity in Fixed and Living Systems

Achieving mutual orthogonal chemistry in vivo remains challenging despite the many successful examples in vitro. For effective in vivo application and potential clinical impact, the reactions need to proceed with high rates and the reactants must be nontoxic and stable under physiological conditions [136, 137]. Biological systems are complex as endogenous biomolecules, enzymes, and reactive species often interfere with bioorthogonal reactions. Strained alkynes, for example, are prone to thiol-ene addition and are less suitable for long intracellular labeling owing to the reductive environment and the presence of high concentrations of free thiols [138]. For labeling biomolecules in vivo, the tetrazine-TCO combination is very prominent owing to its extremely high reaction rates. However, TCOs are liable to isomerization to the inactive cis isomer via light, thermal energy, concentrated thiol solutions, and metalloproteins, such as transcuprein and serum albumin, that contain copper and zinc [139]. These instabilities make the application of strained alkynes and TCOs for intracellular labeling challenging, but not impossible [140]. Improved stability but slower kinetics can lead to higher efficacy, and thus be preferable in vivo, as was shown by Rossin et al. with several variants of TCO [141].

While most click reactions are suitable for use in living systems at low concentration, some catalysts, such as copper needed for CuAAC, are toxic [142]. Copper ligands, such as THPTA, both improve the kinetics and provide protection to oxidation of cell structures [143]. The BTTP and BTTAA-Cu(I) ligand-copper complexes have been used successfully in CuAAC inside *E*. *coli* without toxicity [144]. The low wavelength light used in photoclick chemistry can be cytotoxic. The most toxic conditions are in the UV range [145], although higher wavelengths may also induce photocytotoxicity [146]. The use of low wavelength light is further challenged by the poor skin penetration in vivo [147].

It is evident that using click chemistry in the intracellular environment of living systems is much more challenging compared with fixed cell material. This greatly restricts the use of certain chemistries, thereby limiting the use of multiple bioorthogonal reactions simultaneously. This underscores the need for a more diverse and rapid click reactions that are suitable for the biomolecule labeling in living cells.

## Conclusions

Mutual orthogonal chemistry is a dynamic and transformative chemical and biological research field. It offers immense promise for the future of drug design, imaging techniques, biomaterial and tissue engineering, and precise drug delivery. Despite current limitations and challenges, such as the need for fine-tuned selectivity, adaptation to the complexities of biological environments, and the expansion of substrate scope, the field continues to evolve quickly. Recent advancements in achieving orthogonality through strategies, such as electronic and steric reactant tuning, yielding distinct reaction mechanisms, and induced click chemistry, have advanced mutual orthogonal chemistry to new grounds. The potential for this field to revolutionize drug discovery and expand the understanding of the complex chemical and biological world offers a bright future for its applications.
